# Development and validation of the social frailty scale for the older adult in China

**DOI:** 10.3389/fpubh.2025.1562211

**Published:** 2025-04-02

**Authors:** ChaoMing Hou, XiaoYan Gong, DingXi Bai, WenTing Ji, Huan Chen, XianYing Lu, XinYu Chen, Xiaohui Dong, Jing Gao

**Affiliations:** College of Nursing, Chengdu University of Traditional Chinese Medicine, Chengdu, Sichuan, China

**Keywords:** older adults, social frailty, psychometrics, questionnaire scales, reliability and validity

## Abstract

**Objectives:**

Existing social frailty instruments are not tailored to the linguistic and cultural characteristics of Chinese-speaking patients; a version addressing this gap will increase clinical understanding of their healthcare experience and may help guide social frailty. To develop a Chinese version of a Social Frailty Scale (CVSFS) for the older adult and to examine the psychometric properties of this instrument.

**Method:**

Based on the recommendations of the COSMIN guidelines, the scale development inclued three phases: development of the initial scale, optimisation of scale items, and validation test for scale. The initial CVSFS 1.0 version was developed through literature review, semi-structured interviews, research team discussion, and Delphi method. Then, cross-sectional survey was conducted (*n* = 265) and scale items were optimized based on the survey results using item analysis and exploratory factor analysis (EFA) to form CVSFS 2.0 version. Lastly, the cross-sectional survey (*n* = 287) was repeated using CVSFS 2.0 version, and the reliability and validity of the scale's measurement properties were tested.

**Results:**

The initial scale stage of development formed a 42-item CVSFS 1.0 version. After item analysis and EFA, six items were excluded to form a four-dimension with 36-item CVSFS 2.0 version including individual level, family level, interpersonal level, community and social level. The CVSFS 2.0 version demonstrated good reliability and validity, with a Cronbach's α coefficient of 0.926 and a McDonald's ω estimate of 0.931, split-half reliability of 0.928, and test–retest reliability of 0.978. The I-CVI of the scale was calculated to be 0.889~1.000, and the S-CVI/Ave was 0.930. Confirmatory factor analysis results indicated satisfactory fit indices: χ2/df = 2.17, GFI = 0.813, TLI = 0.932, CFI = 0.937, RMSEA = 0.064.

**Conclusions:**

The CVSFS 2.0 version developed in this study based on a social-ecological framework has high reliability and validity, making it a suitable instrument for evaluating social frailty among the older adult in China.

## 1 Introduction

With the development of social economy, the world population presents a serious aging trend. According to the United Nations report, the number of people aged 60 and over worldwide is expected to rise from 1.08 billion (13.7%) in 2022 to 2.13 billion (22.0%) by 2050 (1). Aging has led to an increase in chronic diseases and health problems which are associated with older people (2). Frailty is an important indicator of healthy aging in older people, resulting in an increased risk of falls, disability and death, and has become a public health priority (3). The World Health Organization defines frailty as “a progressive age-related decline in the physiological system that leads to a reduction in intrinsic capacity stores, it makes a person highly susceptible to stressors and increases the risk of a range of adverse health outcomes” (4). At present, under the circumstance of severe aging, the older adults' social participation, family support, and economic situation are all in a serious state, making it difficult for the older adult to meet the ever-increasing social needs, which easily may trigger an increase in the social frailty of the older adult (5). Social frailty refers to an individual's lack of social resources to meet his or her basic social needs, as well as a lack of social behaviors, social activities and self-management skills (6), which its occurrence may involves aspects in individual, family, as well as societal levels (7, 8). The global prevalence of social frailty in the older adults is 16.8–22.0% reported in meta-analyses, which means that the social fragility is prevalent, indicating that attention should be paid to the health needs of older persons at the social level (9, 10).

Studies have shown that social frailty cause multiple adverse health outcomes in older adults. Psychologically, social frailty is associated with higher levels of stress, anxiety, depression, and poorer emotional regulation (11). Physiologically, the lack of social ability and physical exercise in the older adults with social frailty are easy to cause sarcopenia, which is an important cause of falls in the older adults (12). Additional, social frailty increases the chances of hospital readmission and prolongs the length of stay for older people (13). Moreover, the cohort study conducted in the UK also showed that the risk of death was higher for respondents who had social frailty at the baseline of the survey (14). In summary, the consequences of adverse outcomes caused by social frailty in the older adults are serious. To prevent and ameliorate social frailty in the older adults, providing a convenient social frailty scale with high reliability and validity, as well as wide adaptability is the first step to screen and evaluate the social frailty for the older adults in time.

Unfortunately, accurately diagnosing social frailty and its severity states remains a clinical challenge, hampered by a lack of consensus over the definition and conceptualization of social frailty, with growing numbers of social frailty assessment tools (15–19). At present, there is no gold standard for social frailty assessment, with upward of nine tools including HALFT scale, Social Frailty Comprehensive Questionnaire (SFCQ), Social Frailty Index (SFI), Social Frailty Questionnaire (SFQ), Social Frailty Phenotype (SFP), Social Frailty Screening Index (SFSI), 8-item Social Frailty Scale (SFS-8), Social Vulnerability Ability Index (SVI), and Tilburg Frailty Indicator (TFI), Vulnerability Scale (SFS-8), and Social Vulnerability Index (SVI), but they still have some limitations (20). Firstly, these scales except SVI lack the measurement attribute test, and also can't evaluate the severity of social frailty (21). Secondly, there is a lack of relevant tools in mainland China and also a lack of relevant cross-sectional studies on social frailty of the older adults, which is the gap we found. Among the existing assessment tools, only the HALFT scale (16) and SFCQ (21) were developed based on the Chinese population, of which the HALFT scale does not clearly report the source of the entries, while the SFCQ is the integration of SFQ, SFSI and HALFT belonging to foreign evaluation tools, which lacks sufficient consideration for the local cultural characteristics of China and also without measurement attribute test. As we all know, social frailty of the older adult is influenced by socio-economic environmental factors, including historical background, regional characteristics, cultural background, health literacy, hobbies, and interests (22, 23). Developing localized assessment tools based on the older adult population has wide adaptability in China, which is more conducive to large-scale primary screening and can achieve an effective balance between economy and test efficiency (24).

The Social-Ecological Model (SEM) is a comprehensive framework that recognizes the complex interplay between individuals and their environment, including the individual, interpersonal, community, organizational and broader societal levels. Each level interacts with and influences the others, creating a dynamic system that shapes individual behavior and health outcomes (25). By applying the SEM in the development of social frailty scale, we were able to consider the multiple levels of influence on social frailty and create a comprehensive tool that captures the various dimensions of social frailty among the older adult in China. This approach allowed us to identify key factors at each level that contribute to social frailty and incorporate them into the scale items, ensuring that the scale is both theoretically sound and practically relevant.

In summary, we believe that the multilevel structure of the SEM is an effective analytical model for exploring social frailty factors. Therefore, we innovatively used the SEM Therefore, the purpose of this study is to design a methodologically rigorous and practical Chinese version Social Frailty Scale for the older adult guided by the SEM, to set the diagnostic thresholds and measure its severity states of social frailty. Healthcare administrators and medical practitioners can employ this scale to formulate targeted intervention plans according to the degree of social frailty, aiming to improve the quality of life of the older adult in their later years and promote healthy aging.

## 2 Method

### 2.1 Human ethics and consent to participate declarations

The study of ethics approval was approved (QYYLL-2022-011) by the Ethics Committee of Jinniu People's Hospital of Chengdu (Chengdu, China), and all participants provided informed consent, in adherence to the declarations of Helsinki and Istanbul.

### 2.2 Scale development overview

**Figure 1:**
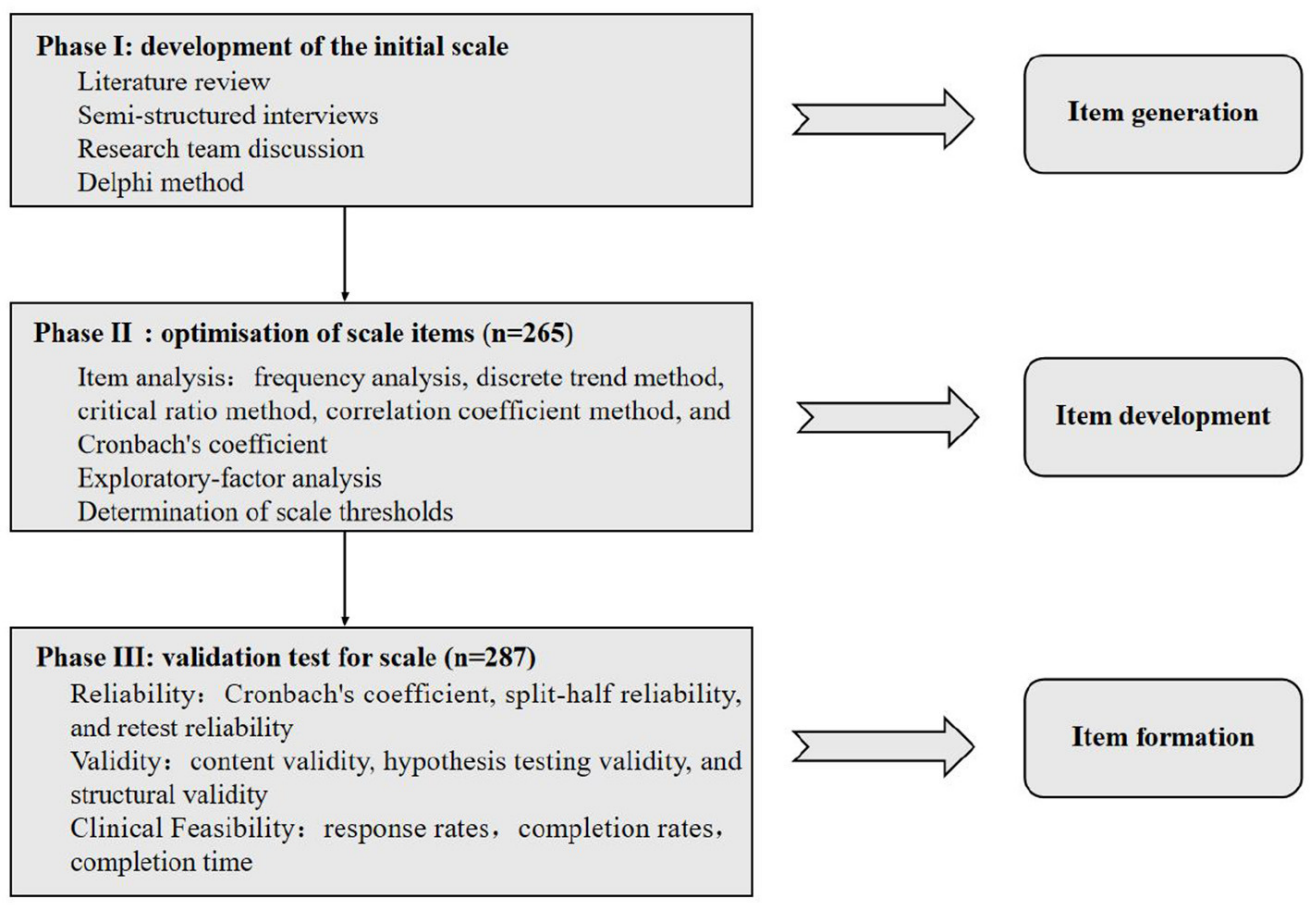
The scale construction process.

The Chinese version of a Social Frailty Scale (CVSFS) for measuring social frailty of older adults was developed in a step-by-step approach. For the development and validation of the questionnaire, we based our methods in line with the criteria stated by the COnsensus-based Standards for selection of health Measurement INstruments [COSMIN; (26–28)]. The COSMIN initiative aims to reach consensus about which measurement properties are considered to be important, their most adequate terms and definitions, and how they should be assessed in terms of study design and statistics (29). The development consisted of the following three phases (30). In Phase I (development of the initial scale), we determined the theoretical framework and constructed the item pool through literature review, semi-structured interviews, and discussion with research team, and then the Delphi method was used to screen and modify the pool of items to form the older adults CVSFS 1.0 version. In Phase II (optimisation of scale items), a formal survey was conducted with the CVSFS 1.0 version among the older adults in the community, and the items were further screened through item analysis and exploratory factor analysis to classify the diagnostic thresholds and severity levels of social frailty to form the older adults CVSFS 2.0 version. Then, in Phase III (validation test for scale), the survey was re-conducted with the CVSFS 2.0 version to test the reliability and validity. The scale construction process is shown in Figure 1.

#### 2.2.1 Phase I: development of the initial scale (item generation)

##### 2.2.1.1 Construct the scale theoretical framework

Within the first step, the goal was to find a theoretical base for the conceptual model of the scale to assess the social frailty for the older adult (31). Models and themes regarding social frailty described in international books, guides, and scientific articles published were identified and discussed by the research team. This research team consists of 10 members, including a chief nurse from the Department of Nursing, an associate chief nurse from the Department of Geriatrics, a physician from the Department of Geriatrics, a medical statistician, two PhDs in Geriatric Nursing, and four current master's degree students in nursing. In addition, they have experience in developing and validating measurement scale.

Consensus was reached, finding the Social-Ecological Model (SEM) as the theoretical framework for the study (25), as the research team found this SEM the best fit with the objective of the study. We selected the SEM because it posits that human development and behavior are influenced by nested individual, interpersonal, community, organizational and broader societal levels of influence, which has been used extensively in public health and health care as an explanatory model and as an intervention framework. The SEM's inclusion of interrelated micro-, meso-, exo-, and macro-level social constructions makes it an apt theoretical framework for exploring nuances of social frailty for the older adult.

##### 2.2.1.2 Create a pool of items

The methods of literature review and semi-structured interviews were used to construct the pool of items. First, a search was conducted in PubMed, Embase, Web of Science, CINAH, CNKI, VIP, Wan fang data, and CBM, from the inception to July 2023, using keywords such as “old/older adult/geriatric” and “social frailty/Social vulnerability/social dimension frailty.” The search yielded a total of 1,166 articles from PubMed, 377 articles from Embase, 546 articles from Web of Science, 172 articles from CINAH, 97 articles from CNKI, 48 articles from VIP, 53 articles from Wan fang data, and 77 articles from CBM. Among these articles, particular attention was paid to the scales related to social frailty, and summarized the influencing factors of social frailty. Details on study selection the process are shown in Appendix 1. Then, we conducted semi-structured interviews to gain a deeper views and experiences of the social frailty from 20 multidisciplinary staff (Appendix 2) in close contact with older adult, served as a supplement to the social frailty dimensions (the interview outline presented in Appendix 3). All interviewees agreed to the whole process being recorded, subsequently research group analyzed the interview results. Finally, the obtained items by literature review and semi-structured interviews were discussed by the research team until consensus was reached on content and objective. The outcome of this step was a finally form a pool of scale items.

##### 2.2.1.3 Form a first draft of the scale

Delphi expert consultation was used to modify and improve the pool of items (32). In this study, a two-round Delphi expert was conducted by e-mail to screen the items, and the target experts were found through peer recommendation and literature search. The expert consultation questionnaire consists of two main parts, the first part is the overall introduction of the study and instructions for completing the form, and the second part is the expert consultation form presenting the dimensions of the scale, the items, the evaluation area, and the suggestion area. The number of experts to be consulted initially for this study is 20. The inclusion criteria of the consulting experts in this study were as follows, a: relevant experts engaged in the direction of geriatric clinical medical care, geriatric community medical care, geriatric psychology, social psychology, etc. b: engaged in the professional field for more than 5 years; c: with a master's degree or above; d: with intermediate or above title in professional technology; e: agreed to participate in the second round of the study. The expert consultation result is determined by the expert authority coefficient (Cr), and expert judgment criteria. The Cr was calculated as follows: Cr = (Ca + Cs)/2 (Ca referring to the expert's judgment criteria for the indexes, and Cs referring to the familiarity degree for the indexes), and Cr >0.7 indicates a high level of authority in the expert consultation. The expert judgment criteria is evaluated using the coefficient of variation and Kendall's W coefficient, where the W is 0~1, and a larger W value indicates better coordination of opinions (33). If the coefficient of variation >0.25, the expert coordination degree of this indicator is considered insufficient. The consultation was terminated when the experts' opinions converged, and the items were revised and deleted based on the statistical results of the experts' opinions, and then the older adults CVSFS 1.0 version was developed.

#### 2.2.2 Phase II: optimisation of scale items (item development)

In this study, the cross-sectional design method was used to optimize the scale items, and the CVSFS 1.0 version was developed to investigate the older adult recruited from 12 communities in western China. Convenience sampling was employed. The inclusion criteria were as follows: (a) older adults ≥60 years of age; (b) no mental illness and normal communication skills; (c) voluntary participation in the study. The exclusion criteria were: (a) participate in other clinical studies; (b) unable to participate in the study. Since the full psychometric validation of scales requires five to 10 participants per item and the older adults CVSFS 1.0 version has 42 items, we aimed to recruit 210 to 420 participants (34). Screening of items using item analysis and exploratory factor analysis based on cross-sectional survey results.

##### 2.2.2.1 Item analysis

We applied frequency analysis, discrete trend method, critical ratio method, correlation coefficient method, and Cronbach's coefficient method to screen the items to ensure the quality of the selected items (35, 36). Appendix 4 provided a detailed operation process. These five methods evaluate and screen the items from different perspectives respectively. A comprehensive judgement is made based on all the statistical results, and the items that meet the inclusion criteria are finally retained. The principle of screening items in this study is that, if an item was excluded by two or more methods, the item would be deleted from the scale. On the contrary, the item would be included if there are at least three methods prompted to be retained.

##### 2.2.2.2 Exploratory-factor analysis

We used exploratory factor analysis to assess the structure of the scale (37). The Bartlett sphericity test and the Kaiser-Meyer-Olkin (KMO) test were used to determine whether factor analysis could be carried out. The Bartlett sphericity test yielded a *P* < 0.05, suggesting that it was suitable for factor analysis. KMO values vary from 0 to 1, and values >0.5 are acceptable (38). Exploratory factor analysis was performed on all items using the maximum variance method of principal component factor analysis. Cumulative contribution >60% suggests an acceptable model. The number of extracted public factors was judged based on the size of the eigenroot of each public factor and the fragmentation plot. The main reference for the retention or deletion of the question items in this study was based on the following: a. Deletion of the items with the largest factor loading < 0.4 in the factor analysis; b. Deletion of the items with the difference between the largest factor loading and the second largest factor loading < 0.2; and c. Deletion of this common factor and the items if there were the number of items under the common factor < 3 (31).

##### 2.2.2.3 Determination of scale thresholds

Diagnostic thresholds can help distinguish whether an older person is social frailty. The survey and validation of the scale we developed were conducted in China. The HALFT scale, which was developed based on the Chinese population and possesses diagnostic thresholds, is currently widely used for screening social frailty. Therefore, this study employs the HALFT scale as the reference threshold for diagnosing of social frailty, and the diagnostic thresholds of this scale were determined using the Receiver Operator Characteristic (ROC) curve (39). Generally, AUC>0.5 is considered diagnostic, whereas an AUC of 1.0 indicates a perfect diagnostic test, 0.85–0.95 is good, 0.7–0.85 is fair, and 0.5–0.7 is considered poor diagnostic accuracy (39). After determining the diagnostic thresholds, the severity of social frailty older adults was graded using the percentile method, with P0~P25 as mild, P25~P75 as moderate, and P75~P100 as severe, respectively. To this point, the older adults CVSFS 2.0 version was developed.

#### 2.2.3 Phase III: validation test for scale (item formation)

We conducted a formal survey of older adults using the CVSFS 2.0 version to assess the measurement properties, including the items' reliability, validity, and feasibility. Using the same inclusion and exclusion criteria as above in the optimisation step of the scale items, study participants were enrolled from 12 communities in western China, and the minimum sample size for factor analysis is 200 participants, consistent with methodological recommendations for factor analysis, which typically suggests a minimum sample size of 10 times the number of questionnaire items to ensure adequate power (40). The questionnaire collected demographic information and contained 36 initial CVSFS 2.0 version. Our study used Cronbach's coefficient, McDonald's omega, split-half reliability, and retest reliability to examine the reliability of the scale, while content validity, hypothesis testing validity, and structural validity to evaluate the validity of the scale (41), as described in Table 1. The feasibility of the CVSFS 2.0 version can be evaluated using the response rate, completion rate, and average completion time. For this, the response rate and completion rate should be >85%, and the average completion time should be < 20 min (44).

**Table 1:** Statistical methods for scale reliability and validity tests.

**Statistical methods**	**Specific operational description**
Cronbach's coefficient	This method indicates the internal correlation and homogeneity between the items of the scale, with a larger alpha representing a better correlation between the items. An acceptable internal consistency is ensured with a coefficient >0.7 (42).
McDonald's omega	Like Cronbach's coefficient, McDonald's ω is a coefficient to estimate the internal consistency reliability. It is generally considered to be more accurate and robust estimate of reliability than Cronbach's alpha, and a value of at least 0.70 can be considered to be acceptable reliability (43).
Split-half reliability	When calculating split-half reliability coefficient, we divided the items into two equal parts with odd and even items and the simple correlation coefficient (r) was calculated for the two-part scores. The Spearman-Brown formula was used for correction when the two scores had the same mean and standard deviation; otherwise, the Rulon formula was used for correction. A split-half reliability >0.7 is generally considered to represent good stability (36).
Test–retest reliability	Retest reliability means that the reproducibility of a measure repeated twice for the same participant, the results of the two scores before and after for each item should be correlated, or else it is considered to be deleted. In this study, 50 of the previous respondents were selected to fill out the same questionnaire again after 14 days, and the data of the two times were evaluated by applying Pearson's coefficient. The correlation coefficient >0.75 indicated that the retest reliability was good.
Content validity	Content validity was evaluated from the perspective of experts and survey respondents, respectively. For the content validity from the experts' perspective, we invited nine experts who had not participated in the previous expert consultation, who had worked in the related fields for more than 5 years and had titles of intermediate level or above, to evaluate the relevance or representativeness of each entry to the dimension to which it belonged (one being irrelevant, two being relevant, three being fairly relevant, and four being highly relevant), and calculated the Scale content validity index, which is generally considered that S-CVI/Ave ≥ 0.9 represents a good content validity (36). Content validity of the participant perspective was assessed qualitatively by inviting ≥50 older adults assessed as social frailty in the survey to give their opinion on whether each entry was relevant, comprehensive, and understandable.
Hypothesis testing validity	Hypothesis test validity, usually expressed as the correlation coefficient between the scores of two scales, is used to measure the agreement between measurements. The HALFT scale was used as a control measurement tool. Our study applied Pearson correlation analysis to explore the correlation between the Social Frailty Assessment Scale for the older adults and the HALFT Scale. Correlations were judged based on correlation coefficients, with >0.5 considered a strong correlation, 0.3 to 0.49 a moderate correlation, and < 0.3 a weak correlation (41).
Structural validity	Structural validity was evaluated using confirmatory factor analysis (CFA). Confirmatory factor analysis is the use of the statistical idea of structural equation modeling to fit a specific factor model with actual data, Amos 27.0 software was used for statistical analysis in this study. CFA employs model fitting, where a model is considered well-fitted if the model's chi-square degrees of freedom (χ2/df) fall within the range of 1–3, goodness-of-fit index (GFI) is ≥0.80, Tucker-Lewis index (TLI) is ≥0.90, comparative fit index (CFI) is ≥0.90, and root mean square error of approximation (RMSEA) is < 0.08 (41).

## 3 Results

### 3.1 Phase I: development of the initial scale

#### 3.1.1 Create a pool of items

According to the initially constructed theoretical framework, after discussing by the research team about the obtained items by literature review and semi-structured interviews, a 45 scale items was drafted, including the individual level (12 items), family level (8 items), interpersonal level, i.e., among peers and colleagues (16 items), and community and social level (9 items; Appendix 5).

#### 3.1.2 Form a first draft of the scale

Initially, we conducted two successive expert consultations. In the first consultation, we provided 20 experts with online questionnaires, and 18 experts responded. The expert positive coefficient was 90%, the expert authority coefficient was 0.892, and the expert coordination coefficient was 0.21 (χ2 = 166.713, *p* < 0.05). Based on the results of the expert consultation and panel discussion, we removed nine items, merged modified 12 items, added six items. Eventually, we formed 42 items. In the second consultation, we provided 18 experts with online questionnaires, and all 18 experts responded. The expert positive coefficient was 100%, the expert authority coefficient was 0.892, and the expert coordination coefficient was 0.278 (χ2 = 168.633, *p* < 0.05). Based on the expert consultation and panel discussion results, we modified eight items. The average age of 18 experts is 19 42.72 (±10.33) years, in the fields of geriatric clinical medicine, mental health, social psychology, public health, and geriatric nursing, whose general information is detailed in Appendix 6. After two rounds of counseling, the experts' opinions converged, so the counseling was terminated. Finally, we retained a 4-dimensional, 42-item CVSFS 1.0 version, including the individual level (12 items), family level (8 items), interpersonal level, i.e., among peers and colleagues (12 items), and community and social level (10 items; Appendix 7).

### 3.2 Phase II: optimisation of scale entries

We distributed 283 questionnaires and received 265 responses (response rate: 93.64%). The data from 265 participants were analyzed, included 115 males and 150 females, with an average age of 71.89 ± 7.04 years, whose general information is detailed in Table 2.

**Table 2:** Demographic characteristics of participants (*n* = 265).

**Variables**	**Categories**	***N* (%)**	**Variables**	**Categories**	***N* (%)**
Sex	Male	115 (43.40)	Economic sources	Retirement pension	122 (46.04)
	Female	150 (56.60)		Governmental subsidy	57 (21.51)
Age	60~69	113 (42.64)		Support by children	68 (25.66)
	70~79	94 (35.47)		Other	18 (6.79)
	≥80	58 (21.89)	Monthly income	< 1,000 RMB	44 (16.60)
Marital status	Single	9 (3.40)		1,000~3,000 RMB	103 (38.87)
	Married	177 (66.79)		3,000~5,000 RMB	89 (33.58)
	Divorced	36 (13.58)		>5,000 RMB	29 (10.94)
	Widow	43 (16.23)	Living situation	Alone	44 (16.60)
Education level	Primary and below	69 (26.04)		With children	40 (15.09)
	Middle school	78 (29.43)		With spouse	108 (40.75)
	High school	87 (32.83)		With spouse's children	31 (11.70)
	College and above	31 (11.70)		Other	42 (15.85)

#### 3.2.1 Item analysis

According to the various statistical results of the item analysis (Appendix 8), the frequency analysis and critical ratio methods showed that no items were removed; the discrete trend method showed that item 7, 10, and 30 had an SD < 0.8 and were considered for deletion; and the correlation coefficient method showed that the correlation coefficients of item 7, 10, 15, 21, 26, 30, 37, and 38 with the total scores were < 0.4, and they were considered for deletion; the Cronbach's coefficient method showed that the Cronbach's α of the scale increased after the exclusion of item 10, 26, 30, 37, and 38, which were considered for deletion. Item 7, 10, 26, 30, 37, and 38 were deleted because two or more items in the item analysis did not meet the criteria. As a result, the CVSFS 2.0 version was constructed with four dimensions and 36 items, which is shown in Appendix 9.

#### 3.2.2 Exploratory-factor analysis

**Figure 2:**
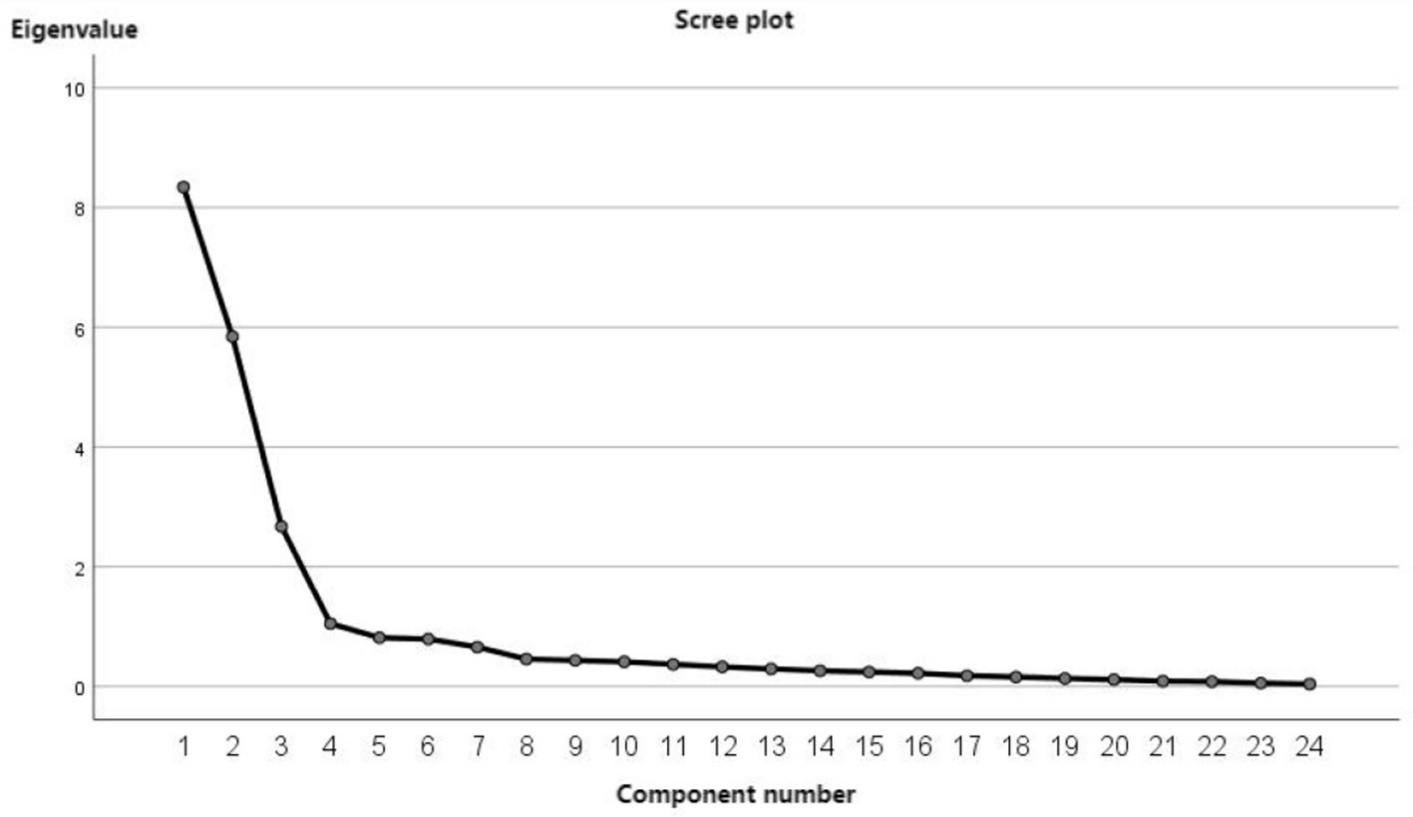
Exploratory factor analysis gravel plot.

The scale entries analyzed by items were subjected to exploratory factor analysis. The validity was assessed through KMO and Bartlett's test of sphericity. The KMO value was 0.908, *p* < 0.001, which is >0.60, and Bartlett's test of sphericity showed high significance (χ2 = 9860.838, df = 630, *p* < 0.01), indicating the presence of common factors among variables, making the data suitable for factor analysis. Principal component analysis and maximum variance rotation were used and extracted four factors with characteristic root values >1, which could explain 70.460% of the cumulative variance, and the fragmentation diagram is shown in Figure 2. The rotated component matrix revealed that the factor internal loadings of each entry were all >0.4 and the factor coefficients differed by >0.2 in two and more factors, so all 36 entries were retained (Table 3).

**Table 3:** Factor loadings of the CVSFS 2.0 version (*n* = 265).

**Items**	**Factor loading (4-factor model)**			
	**Individual level**	**Family level**	**Interpersonal level**	**Community and social level**
1	**0.961**	0.010	0.044	0.111
2	**0.918**	−0.003	0.026	0.103
3	**0.783**	0.051	−0.067	0.085
4	**0.873**	−0.014	0.010	0.081
5	**0.770**	0.086	−0.018	0.057
6	**0.904**	−0.018	−0.004	0.133
7	**0.890**	0.003	0.036	0.008
8	**0.889**	0.062	0.106	0.078
9	**0.931**	−0.038	0.029	0.148
10	**0.953**	−0.008	0.014	0.121
11	0.148	0.064	−0.060	**0.744**
12	0.127	0.133	0.053	**0.805**
13	0.301	−0.076	−0.135	**0.623**
14	0.007	0.214	0.157	**0.834**
15	0.073	0.123	0.196	**0.650**
16	0.133	0.101	0.159	**0.791**
17	0.049	0.036	0.411	**0.619**
18	0.015	0.136	0.257	**0.768**
19	−0.010	**0.551**	−0.098	0.001
20	0.074	**0.823**	0.275	0.159
21	0.081	**0.736**	0.293	0.105
22	0.006	**0.774**	0.171	0.106
23	0.024	**0.788**	0.388	0.143
24	−0.099	**0.594**	0.201	0.104
25	0.021	**0.797**	0.293	0.096
26	0.057	**0.841**	0.360	0.118
27	0.034	**0.835**	0.283	0.077
28	0.012	**0.836**	0.132	0.026
29	0.028	0.313	**0.884**	0.146
30	−0.005	0.192	**0.827**	0.090
31	−0.007	0.168	**0.816**	0.111
32	0.030	0.231	**0.773**	0.202
33	0.023	0.252	**0.829**	0.064
34	0.018	0.172	**0.638**	0.069
35	0.046	0.215	**0.751**	0.059
36	−0.025	0.263	**0.888**	0.139

Bold indicates the highest factor loading for each item.

#### 3.2.3 Determination of scale thresholds

**Figure 3:**
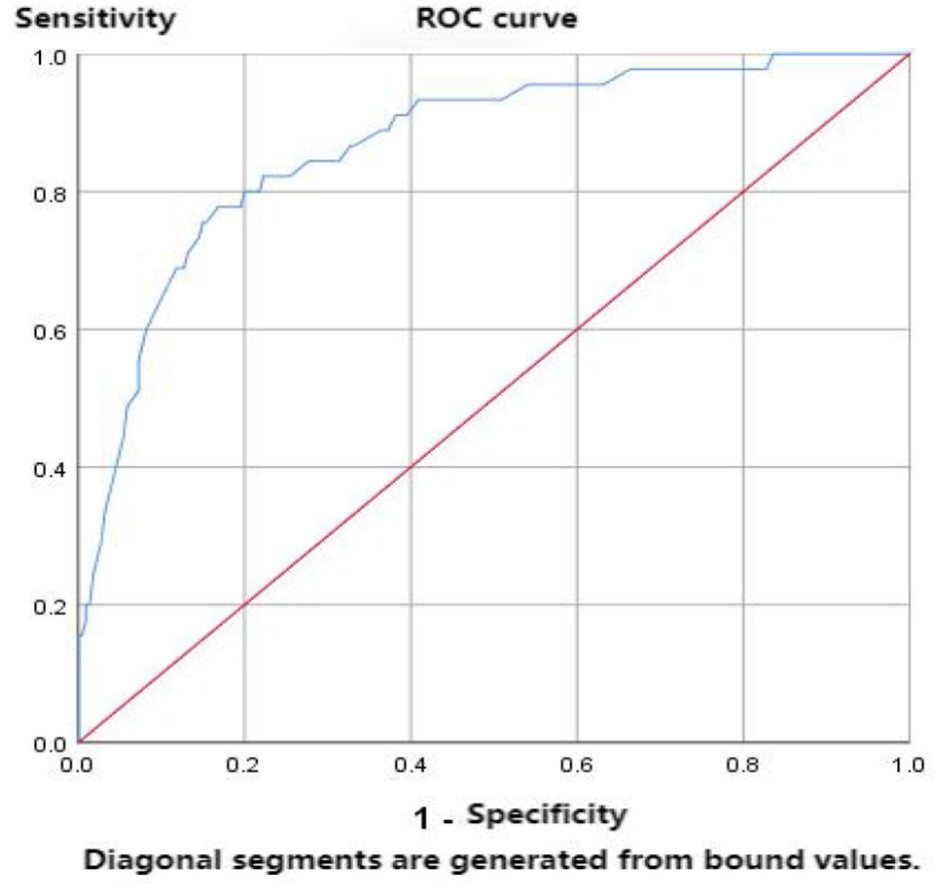
ROC curve diagram.

We used the ROC curve to help determine the diagnostic threshold of this scale, and the results showed that the area under the ROC curve was 0.868, *P* < 0.001, indicating that this scale has good accuracy for assessing social frailty, as shown in Figure 3. The highest value of the Youden Index (YI) in this study was 0.61, with a sensitivity of 77.8% and a specificity of 83.2%. The statistical results showed a diagnostic threshold of 115.5 points for the scale, but since the entry scores were all integers, the diagnostic threshold was determined to be 116, which means that ≥116 points were assessed as socially debilitating (Appendix 10). The results of defining the assessment level of the scale using the percentile method are as follows: 116 ≤ score ≤ 121 represents mild severity of social frailty in the older adults; 122 < score ≤ 135 represents moderate severity; and score > 135 represents severe severity.

### 3.3 Phase III: validation test for scale

We distributed 300 questionnaires and received 287 responses (response rate: 95.67%). The data from 287 participants were analyzed, included 138 males and 149 females, with an average age of 72.67 ± 8.12 (60~89) years, whose general information is detailed in Table 4.

**Table 4:** Demographic characteristics of participants (*n* = 287).

**Variables**	**Categories**	***N* (%)**	**Variables**	**Categories**	**N (%)**
Sex	Male	138 (48.08)	Economic sources	Retirement pension	121 (42.16)
	Female	149 (51.92)		Governmental subsidy	52 (18.12)
Age	60~69	121 (42.16)		Support by children	68 (23.69)
	70~79	96 (33.45)		Other	46 (16.03)
	≥80	70 (24.39)	Monthly income	< 1,000 RMB	25 (8.71)
Marital status	Single	12 (4.18)		1,000~3,000 RMB	126 (43.90)
	Married	201 (70.03)		3,000~5,000 RMB	99 (34.49)
	Divorced	35 (12.20)		>5,000 RMB	37 (12.89)
	Widow	39 (13.59)	Living situation	Living alone	42 (14.63)
Education level	Primary and below	55 (19.16)		Living with children	60 (20.91)
	Middle school	118 (41.11)		Living with spouse	122 (42.51)
	High school	74 (25.78)		Living with spouse's children	30 (10.45)
	College and above	40 (13.94)		Other	33 (11.50)

#### 3.3.1 Reliability tests

The Cronbach's α coefficient for the CVSFS 2.0 version in the older adults Scale 2.0 was 0.926, and the Cronbach's α coefficients for the dimensions ranged from 0.891 to 0.972 exceeding 0.7, meanwhile, McDonald's ω coefficient for the total scale was 0.931, and those for the four dimensions ranged from 0.913 to 0.976, which indicates that the internal consistency of the scale is good. Spearman-Brown correlation coefficients were computed for items divided into two equal parts randomly, showing that the split-half coefficients with dimension scores were ranged from 0.865 to 0.964 exceeding 0.7, with a correlation coefficient of 0.928 with the total scale score. In this study, 50 previous respondents were selected to fill out the same questionnaire again after 14 days, and the data of the two times were evaluated. The test-retest reliability of the scale was 0.978, and the re-test reliabilities of the dimensions ranged from 0.931 to 0.980, which were statistically significant (*P* < 0.01), indicating that the stability of the scale was good (Table 5).

**Table 5:** Reliability coefficients of the total scale and each domain in CVSFS 2.0 version (*n* = 287).

**Dimensions**	**Items**	**Cronbach's α**	**McDonald's omega**	**Split-half reliability**	**Test-retest reliability (*n* = 50)**
Overall scale	36	0.926	0.931	0.928	0.978
Individual level	10	0.972	0.976	0.964	0.974
Family level	8	0.891	0.913	0.865	0.931
Interpersonal level	10	0.939	0.950	0.934	0.980
Community and social level	8	0.941	0.952	0.925	0.976

#### 3.3.2 Validity tests

**Figure 4:**
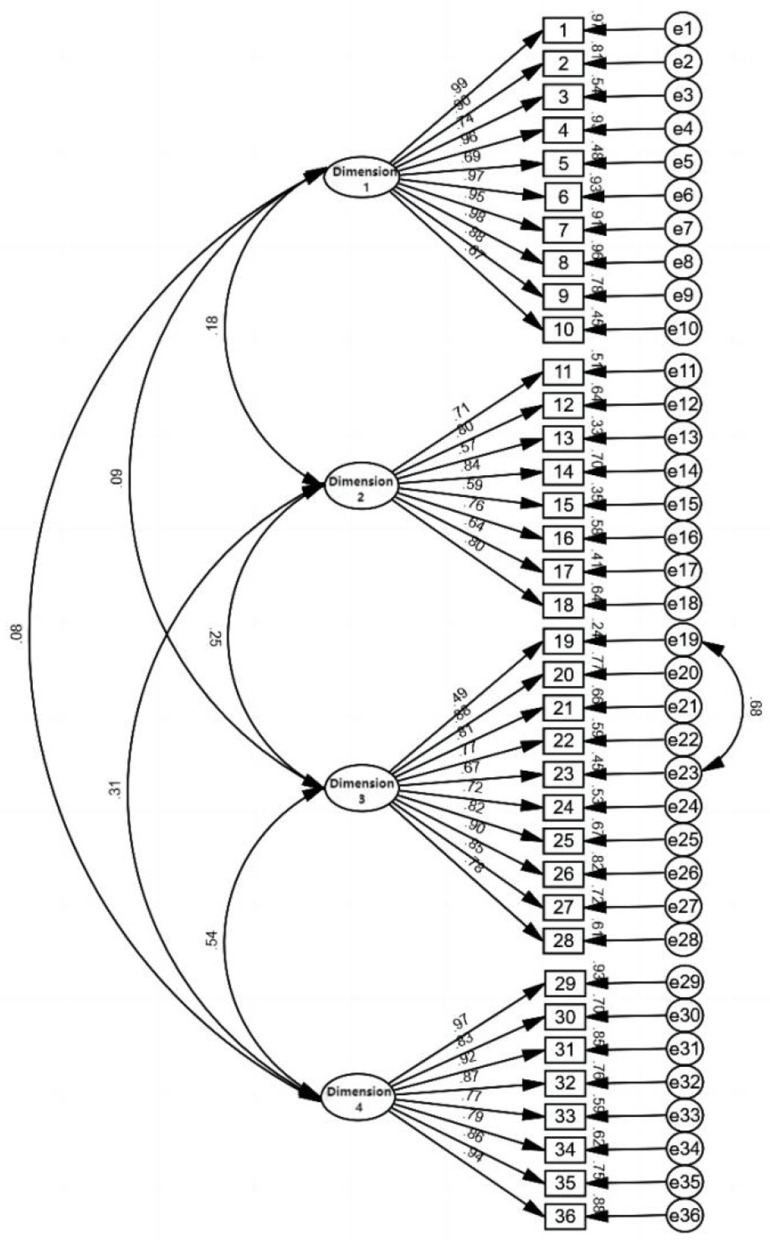
Modified structural equation model diagram of CVSFS 2.0 version for the older. Dimension 1, individual level; Dimension 2, family level; Dimension 3, interpersonal level; Dimension 4, community and social level. Numbers 1~36 are scale items 1~36; e1~e36 are residuals.

The content validity counted the results of the correlation scores of nine experts for each item, and the I-CVI of the scale was calculated to be 0.889~1.000, and the S-CVI/Ave was 0.930 (Appendix 11), while the proportion of positive opinions on the content validity from the 50 participants' perspectives was >94% (Appendix 12), which indicated that the content of the scale could truly reflect the purpose of the measurement. The results of the Pearson correlation analysis used for hypothesis testing validity revealed a significant correlation coefficient of 0.512 between the total scores of the CVSFS 2.0 version and the HALFT Scale, with a *P* < 0.001, which is a strong correlation, indicating that the scale has a good diagnostic efficacy. The correlation coefficients between the scores of each dimension of the CVSFS 2.0 version and the HALFT Scale ranged from 0.301 to 0.342 (*P* < 0.001), suggesting a moderate correlation. This indicates that while there is a relationship between the scores of each dimension and the total score of the HALFT Scale, there are also distinct differences. For detailed results (see Table 6). The 287 data points from the formal survey were subjected to CFA calculations, GFI did not meet the standard requirements, revealing suboptimal model fit [χ2/df =2.456, GFI = 0.789, TLI = 0.916, CFI = 0.922, RMSEA = 0.071] (Appendix 13). To enhance model selection, potential modifications were considered, employing some recommended Maximum Likelihood estimation-based corrections (MIs) to reduce cross-loadings. Through iterative adjustments, the modified model eventually encompassed 36 items across four factors, exhibiting the following fit indices: χ2/df = 2.17, GFI = 0.813, TLI = 0.932, CFI = 0.937, RMSEA = 0.064, and the fitness indicators met the criteria, indicating good structural validity of the scale (Table 7, Figure 4).

**Table 6:** Correlation analysis of CVSFS 2.0 version and the HALFT scale.

**CVSFS 2.0 version**	**HALFT total score**	**P**
Overall scale score	0.512	< 0.001
Individual level score	0.342	< 0.001
Family level score	0.301	< 0.001
Interpersonal level score	0.319	< 0.001
Community and social level score	0.335	< 0.001

**Table 7:** Goodness-of-fit of the CVSFS 2.0 version for the older model (*n* = 287).

	***χ^2^*/*df***	**GFI**	**TLI**	**CFI**	**RMSEA**
Recommended range	1~3	≥0.800	≥0.900	≥0.900	< 0.080
Initial model	2.456	0.795	0.916	0.922	0.071 (0.067, 0.076)
Adjusted model	2.170	0.813	0.932	0.937	0.064 (0.059, 0.069)

#### 3.3.3 Clinical feasibility

The response rates and completion rates of the CVSFS 2.0 version were 95.67%, 100%, respectively. The completion time was 10~15 min.

## 4 Discussion

### 4.1 Main findings

Social frailty is an important social health problem faced by the older adult, and health policy makers and medical practitioners should assess the current status of the occurrence of social frailty in the older adult, and then target the development of social frailty improvement measures. Assessing social frailty in older adults provides a comprehensive understanding of the social dimensions of health, therefore we developed and validated a new frailty-specific instrument for the older adult, the Chinese version of a Social Frailty Scale (CVSFS) based on the theoretical framework of SEM, as a valid assessment tool for assessing social frailty in older adults in China, resulting four dimensions and 36 items.

SEM suggests that human development is a process of interaction at all levels, emphasizing the interaction between the organism and the environment. In the 4-level nested structure proposed by SEM, the micro-system can be understood as the direct environment in which the individual lives, such as the individual's subjective emotions, level of self-care, and patterns of daily living; the meso-system describes the connections and processes between micro-systems, such as family support, and relationships between family members; the external system includes the connections between environments that occur to indirectly influence the individual, such as neighborhood relationships, social interactions, and social activity participation, and macro-systems refer to the influence of the social environment and socio-human factors on the individual, such as community experience, cultural inclusiveness, and sense of social belonging, and the use of the theory allows for an in-depth discussion of social frailty at all levels (45). Under the guidance of SEM, the present study established four dimensions of the scale based on the micro-system, meso-system, external system and macro-system, namely, individual level, family level, interpersonal level, and community and social level, which comprehensively cover the influence of the environment on the older adult at all levels. The occurrence of social frailty in the older adult is affected by the degree of interaction between the older adult themselves and the outside world. The philosophical viewpoints expressed in this theory are consistent with the practical needs of this study, and it covers the influence of the environment on the older adult at all levels in a more comprehensive way, which is a good guide for the development of the social frailty scale. After the optimisation of the scales and the reliability test, the scales developed in this study based on the theory performed well, indicating that the content of the items fit the theoretical framework.

The HALFT scale, as a control tool in this study, is a screening tool with fewer items and lacks the steps to test the theoretical basis and the structure of the measure, and the scientific validity of the research application is limited (16). The remaining social frailty assessment scales also have limitations in the development/translation process, such as insufficient theoretical basis, poor content validity design and lack of statistical steps, and their authenticity and reliability still need to be verified (46). In addition, the existing social frailty assessment tools do not set diagnostic thresholds and severity levels and are not applicable to the Chinese socio-cultural context, which has certain limitations in application, which restricts the development of research in the field of social frailty in China (47). Compared with the existing social frailty instruments, the social frailty assessment tool developed in this study strictly follows the scale development process, is based on theoretical concepts, has more complete measurement test results, and the reliability results show that it is highly feasible and operable in the Chinese region.

Social frailty in older adults is a dynamic and reversible process that can be ameliorated through timely identification and intervention (48). It is known that the causes of social frailty in older adults are numerous, covering social support, family functioning, economic level, and psychosocial factors. Consequently, improving social frailty requires the participation of the older adult themselves, their families, and social organizations, which this collaborative approach is instrumental in enhancing the wellbeing and quality of life of older adults (49). Different types of interventions are applicable when the severity of social frailty in older adults varies. When the degree of social frailty is mild, health guidance, participation in social activities, and increased verbal communication can delay or improve social frailty (50); whereas, when the degree of social frailty is severe, more systematic health services, such as home-delivered meals, home- and community-based services, or specialized psychotherapy are required to promote the social integration of older people (6). The scale developed in this study can not only help healthcare workers to identify older adults with social frailty in a timely manner, but also assess the severity of social frailty in older adults, so that healthcare resources can be allocated rationally according to the degree of social frailty, laying the foundation for the development of a more targeted intervention programme for social frailty in older adults in the future, and enhancing older adult's social participation and sense of belonging to the society (51).

Relative to other similar studies (15–19, 52), this study further expands the application of social ecology, proving the adaptability of social ecology theory combined with different subject studies, which also provides a basis for future studies to continue exploring the theoretical implications of social ecology in older research. On the other hand, we provide a comprehensive research perspective on the study of social frailty in older adults. Both in terms of measurement instruments and theoretical research, we argue that the sources of support are multidimensional. Analysis, measurement, and intervention of social frailty in older adults should begin at multiple levels, and any single level of intervention or measurement may be one-sided. In terms of measurement instrument development, it is clear that the CVSFS is a new measurement instrument that can be used to measure Chinese olders' social frailty, filling a gap in localized measurement instruments.

### 4.2 Practical implications

The Chinese version of a Social Frailty Scale (CVSFS), as the first practical scales developed specifically for China's sociocultural context with rigorous methodology, scientific steps and comprehensive reports. The CVSFS can serve as a rapid screening tool in primary care clinics and community health centers to identify socially frail older adults, enabling targeted interventions (e.g., social engagement programs, family support coordination). Furthermore, it can be used in tertiary medical institutions to evaluate social frailty severity and guide personalized care plans. To ensure its widespread use, it is necessary to organize training courses and workshops for healthcare professionals, community workers, and researchers, providing them with practical guidance and support, and also collaborate with professional organizations like the Chinese Geriatrics Society and the Chinese Association of Gerontology and Geriatrics to promote its application and popularization, thus playing a positive role in the care and health management of the older adult in China.

### 4.3 Limitations

Convenience sampling was used in this study, and the developed scale is currently only validated in a part of the older adults population in some communities in western China. Considering the great differences in the living habits and physical conditions of the older adults in different regions, it should be tested in a larger population before being promoted for use in the future. In addition, when choosing the research subjects for qualitative interviews, considering the academic nature of the concept of social infirmity and the difficulty of the older adults to understand the situation, we chose the staff of related professions to conduct the interviews, and the staff's personal opinion tendency and the degree of understanding of the research content may lead to a certain degree of bias in the results of the interviews.

## 5 Conclusion

This study developed and validated a Chinese version of a social frailty instrument, comprising 36 items in four subscales related to individual level, family level, interpersonal level, and community and social level. The instrument demonstrated favorable reliability and validity and serves as a useful tool for health professionals to monitor treatment progress via patient- reported social frailty. Further validation incorporating larger sample sizes and various geographical locations is warranted.

## Data Availability

The original contributions presented in the study are included in the article/Supplementary material, further inquiries can be directed to the corresponding authors.
