# Supplementary material for: Development and validation of the social frailty scale for the older adult in China
Source: Front Public Health. 2025 Apr 2;13:1562211. doi: 10.3389/fpubh.2025.1562211 (PMC12013530; doi:10.3389/fpubh.2025.1562211)
Supplement: Supplementary file 1 [file Table_1.docx]

# Appendix 1


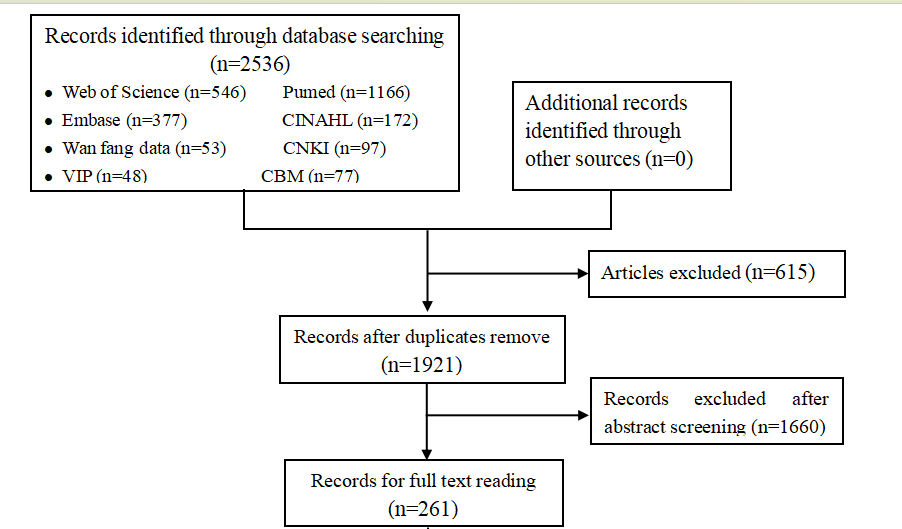


# Appendix 2

| Basic information of the 20 multidisciplinary staff through semi-structured interviews | | | | | | |
| --- | --- | --- | --- | --- | --- | --- |
| Serial number | Fields of work | Education background | Professional title | Age | Gender | Working experience |
| P1 | Applied Psychology | Undergraduate | Middle level | 36 | Male | 10 |
| P2 | Applied Psychology | Undergraduate | Junior | 29 | Female | 6 |
| P3 | Geriatric Mental Health | Master | Senior | 60 | Female | 26 |
| P4 | Geriatric Mental Health | Undergraduate | Junior | 27 | Female | 6 |
| P5 | Social Medicine and Health Care Management | Doctor | Senior | 55 | Female | 24 |
| P6 | Social Medicine and Health Care Management | Master | Middle level | 39 | Male | 8 |
| P7 | Public Health | Undergraduate | Junior | 28 | Male | 7 |
| P8 | Public Health | Undergraduate | Junior | 26 | Female | 5 |
| P9 | Gerontological Nursing | Doctor | Senior | 50 | Female | 20 |
| P10 | Gerontological Nursing | Master | Middle level | 38 | Female | 12 |
| P11 | Gerontological Nursing | Master | Junior | 26 | Female | 5 |
| P12 | Gerontological Nursing | Master | Junior | 30 | Male | 5 |
| P13 | Clinical Medicine | Master | Middle level | 41 | Male | 9 |
| P14 | Clinical Medicine | Undergraduate | Junior | 33 | Male | 8 |
| P15 | Clinical Nursing | Undergraduate | Middle level | 38 | Female | 15 |
| P16 | Clinical Nursing | Junior College | Middle level | 32 | Female | 12 |
| P17 | Clinical Nursing | Junior College | Junior | 26 | Female | 5 |
| P18 | Community-based Work | Undergraduate | None | 32 | Male | 7 |
| P19 | Community-based Work | Junior College | None | 45 | Female | 21 |
| P20 | Community-based Work | High School | None | 49 | Male | 18 |

# Appendix 3

| the interview outline |
| --- |
| 1. Do you have contact with social frailty older persons in your work or in your life? Can you describe the exact circumstances? (We hope you can give us some examples of what you have encountered in your work and life.) |
| 1. What are the behaviors of an older person that would enable you to conclude that the older person has a socially frailty condition? |
| 1. Taking the example of an older person close to you who may be experiencing socially frailty, what do you think are the reasons for his/her socially frailty? |
| 1. What emotional problems do you think socially frailty older people may face and what emotional needs do they have? |
| 1. What do you think are the likely social characteristics of a socially frailty older person and how does his/her lack of integration into social groups affect him/her? |
| 1. As the organism declines, the status of the older adult in the family and society declines, what are the specific manifestations of declining status in the lives of socially frailty older adult people you have encounter? |
| 1. In addition to the above, what other behaviors of older people have you seen that are detrimental to older people's social interaction, social participation or the fulfilment of social needs? |
| 1. What else would you add to the above questions? |

# Appendix 4

**Statistical methods for item analysis**

| Statistical methods | Specific operational description |
| --- | --- |
| frequency analysis | 1. Difficulty analysis: expressed in terms of the pass rate of an entry, when there is a part of the respondents did not answer a certain entry, it means that the entry is inappropriate or difficult to understand, consider deleting. The general requirement is a pass rate of ≥80%.   b. Response analysis: indicates the concentration of the respondents' answers to each entry and the validity of the selected items. When an entry's responses are concentrated on one choice item (more than 80%) or a choice item is not answered at all are inappropriate, consider deleting. |
| discrete trend method | The method is to carry out screening from the perspective of entry sensitivity. Standard Deviation (SD) is generally used as a statistical indicator, and the smaller the SD value of an entry, the poorer its ability to differentiate and the less sensitive it is to the differences in survey respondents. Entries with SD <0.8 are considered for deletion. |
| critical ratio method | The total scores of the scale were ranked according to the lowest to the highest, dividing the low subgroups into the top 27% of the rankings and the high subgroups into the bottom 27% of the rankings. The researcher carried out independent samples t-test for the high and low subgroups respectively, if there is no difference between the scores of the two groups, it means that the entries are poorly differentiated, and the entries whose CR values did not reach the level of significance (*P*>0.05) or t-value < 0.3 were considered for deletion. |
| correlation coefficient method | The correlation coefficient method screens evaluation indicators mainly from the perspective of representativeness and independence. In this study, the Pearson correlation coefficient method was applied to calculate the correlation coefficient. The entries with correlation coefficients >0.4 between the retained entries and the total score were selected, and the entries ≤0.4 were deleted. |
| Cronbach's coefficient method | The method screens entries in terms of internal consistency. The Cronbach's alpha coefficient is calculated for a dimension and the change in the coefficient after removing an entry is compared. If there is an increase in the Cronbach's α coefficient after removing an entry, it means that the presence of this entry reduces internal consistency and it is considered for removal. |

# Appendix 5

| The pool of scale items | |
| --- | --- |
| **Dimension** | **items** |
| **individual level** | 1.I've become very reluctant to talk. |
|  | 2.I often feel lonely now. |
|  | 3. I became less willing to do housework. |
|  | 4.I became less concerned with my outward appearance. |
|  | 5. I am not satisfied with my present life. |
|  | 6.I feel like every day is boring. |
|  | 7. I feel like no one cares about me. |
|  | 8.I don't have much to look forward to in the future right now. |
|  | 9. I don't know what I can do every day. |
|  | 10. I don't pay much attention to my health. |
|  | 11. I don't have a hobby that I want to put my energy into. |
|  | 12. My financial situation is not enough to cover the living expenses. |
| **family level** | 13. I always live alone at home. |
|  | 14. My family doesn't visit me often. |
|  | 15. I don't get along too well with my family. |
|  | 16. *My family provides help in a timely manner when I need it (reverse item). |
|  | 1. I usually don't like to bother my family for help. |
|  | 18. I feel that I am not recognized by my family. |
|  | 19. My family always fails to understand my thoughts. |
|  | 20. I spend a lot of time looking after my children and grandchildren. |
| **interpersonal level** | 21. I go out less than I did last year. |
|  | 22. I seldom communicate with others by telephone now. |
|  | 23. I have no one to turn to for advice right now. |
|  | 24. I am not satisfied with my current neighborhood relationships. |
|  | 25. I'm used to being a loner. |
|  | 26. I don't often visit my friends' homes. |
|  | 27. I don't usually participate in social activities on a regular basis (e.g., chess/ square dancing/religious activities, etc.). |
|  | 28. I don't have a social circle (e.g. recreational/fitness group) that I belong to right now. |
|  | 29. My medical condition prevents me from taking part in social activities. |
|  | 30. I'm worried about people gossiping about me. |
|  | 31. I rarely travel long distances by transport now (e.g. train/car). |
|  | 32. I'm less inclined to engage with strangers now. |
|  | 33. I sometimes can't understand what others say. |
|  | 34. I can't express clearly in words what I have in mind. |
|  | 35. My poor hearing makes it difficult for me to communicate with others. |
|  | 36. I would feel tired when dealing with other people now. |
| **community and social level** | 37. I don't feel cared for by social organizations. |
|  | 38. I feel that community services do not adequately take into account the needs of the older adult. |
|  | 39. I am not satisfied with the present social medical service. |
|  | 40.*I feel that social policies provide good living security for the older adult (reverse item). |
|  | 41. I have a hard time understanding new things in society. |
|  | 42. I often fail to understand the minds of young people today. |
|  | 43. I feel like I don't fit in with the current social development. |
|  | 44. I can't use smart electronic devices skillfully. |
|  | 45. I feel my social status has declined. |

| The pool of scale items | |
| --- | --- |
| 维度 | 条目 |
| 个体层面 | 1.我平常不太愿意讲话。 |
|  | 2.我常常感到孤独。 |
|  | 3.我变得很少主动去做家务。 |
|  | 4.我不在意自己的外在形象。 |
|  | 5.我对现在的生活状态不满意。 |
|  | 6.我感觉每天的生活都很无趣。 |
|  | 7.我感觉没有人关心自己。 |
|  | 8.我现在对未来的生活没什么期待。 |
|  | 9.我每天不知道可以做些什么。 |
|  | 10.我不太关注自己的健康状况。 |
|  | 11.我没有愿意投入精力的爱好。 |
|  | 12.我的经济状况不足以承担生活开销。 |
| 家庭层面 | 13.我总是一个人在家中居住。 |
|  | 14.我的家人不常来探望我。 |
|  | 15.我和家人相处不太融洽。 |
|  | 16.*在我需要时家人会及时提供帮助（反向条目）。 |
|  | 1. 我平时不愿意劳烦家人帮忙。 |
|  | 18.我感觉自己不被家人认可。 |
|  | 19.家人总是不能理解我的需求。 |
|  | 20.我很多时间都用来照顾子孙。 |
| 社交层面 | 21.我的外出次数要比去年少。 |
|  | 22.我现在很少用电话和别人联系。 |
|  | 23.我现在没有可以寻求建议的人。 |
|  | 24.我对现在的邻里关系不满意。 |
|  | 25.我平时习惯独来独往。 |
|  | 26.我不常去拜访朋友的家。 |
|  | 27.我不经常参加社会活动（如下棋/广场舞/志愿者/宗教活动等）。 |
|  | 28.我没有属于我的社交圈。 |
|  | 29.我的身体状况造成我无法参与社会活动。 |
|  | 30.我担心别人说关于我的闲言碎语。 |
|  | 31.我现在很少乘坐交通工具长途出行（如火车/汽车）。 |
|  | 32.我不愿意和陌生人接触。 |
|  | 33.我有时不能理解别人讲话的内容。 |
|  | 34.我不能用语言清晰地表达我心中所想。 |
|  | 35.我的听力较差造成我无法与人正常交流。 |
|  | 36.和别人打交道让我感觉很累。 |
| 社会层面 | 37.我感觉不到社会组织对我的关心。 |
|  | 38.我感觉社区服务没充分考虑老年人需求。 |
|  | 39.我不满意现在的社会医疗服务。 |
|  | 40.*我感觉社会政策对老年人很友好（反向条目）。 |
|  | 41.我很难理解社会新兴事物。 |
|  | 42.我经常无法理解现在年轻人的想法。 |
|  | 43.我感觉自己不适应现在的社会发展。 |
|  | 44.我不能熟练地使用智能电子设备。 |
|  | 45.我感觉自己的社会地位下降了。 |

# Appendix 5

| General situation of experts | | | | | | |
| --- | --- | --- | --- | --- | --- | --- |
| Serial number | Gender | Fields of work | Professional title | Education background | Age | Working experience |
| Z1 | Male | Clinical Medicine | Chief physician | Master | 62 | 30 |
| Z2 | Male | Clinical Medicine | Associate chief physician | Doctor | 40 | 18 |
| Z3 | Male | Clinical Medicine | Attending physician | Master | 38 | 15 |
| Z4 | Female | Clinical Medicine | Attending physician | Master | 36 | 12 |
| Z5 | Female | Clinical Medicine | Attending physician | Master | 33 | 8 |
| Z6 | Male | Mental health | Professor | Doctor | 51 | 22 |
| Z7 | Female | Mental health | Associate Professor | Doctor | 49 | 18 |
| Z8 | Female | Mental health | Attending physician | Master | 37 | 9 |
| Z9 | Male | Social psychology | Professor | Doctor | 66 | 32 |
| Z10 | Female | Social psychology | Associate Professor | Master | 55 | 24 |
| Z11 | Female | Social psychology | Associate Professor | Master | 42 | 17 |
| Z12 | Male | Public Health | Lecturer | Master | 35 | 14 |
| Z13 | Male | Public Health | Lecturer | Master | 32 | 5 |
| Z14 | Female | Gerontological Nursing | Professor | Doctor | 50 | 19 |
| Z15 | Female | Gerontological Nursing | Associate Professor | Doctor | 39 | 13 |
| Z16 | Female | Gerontological Nursing | Associate Chief Nurse | Master | 36 | 15 |
| Z17 | Female | Gerontological Nursing | Nurse supervisor | Master | 35 | 11 |
| Z18 | Female | Gerontological Nursing | Nurse supervisor | Master | 33 | 9 |

# Appendix 7: CVSFS 1.0 version

Compared to the past, have you experienced any of the following changes/been in any of the following situations in the last year? Please put a check mark under the options that match.

| **Scale entry** | **Fully compliant** | **Compliant** | **Neutral** | **Not compliant** | **Not compliant at all** |
| --- | --- | --- | --- | --- | --- |
| **Dimension 1: Individual level** | | | | | |
| 1.I've become very reluctant to talk. | 5 | 4 | 3 | 2 | 1 |
| 2.I often feel lonely now. | 5 | 4 | 3 | 2 | 1 |
| 3.I don't have the energy to take care of my daily routine. | 5 | 4 | 3 | 2 | 1 |
| 4.I became less concerned with my outward appearance. | 5 | 4 | 3 | 2 | 1 |
| 5.I often feel overwhelmed and have a sense of uselessness. | 5 | 4 | 3 | 2 | 1 |
| 6.I feel like every day is boring. | 5 | 4 | 3 | 2 | 1 |
| 7.I feel like no one cares about me. | 5 | 4 | 3 | 2 | 1 |
| 8.I can't control my emotions sometimes. | 5 | 4 | 3 | 2 | 1 |
| 9.I don't have much to look forward to in the future right now. | 5 | 4 | 3 | 2 | 1 |
| 10.I don't know what I can do every day. | 5 | 4 | 3 | 2 | 1 |
| 11.I lost my enthusiasm for my former hobbies. | 5 | 4 | 3 | 2 | 1 |
| 12.I think financial hardship is a major factor in my standard of living. | 5 | 4 | 3 | 2 | 1 |
|  | | | | | |
| **Dimension 2: family level** | **Fully compliant** | **Compliant** | **Neutral** | **Not compliant** | **Not compliant at all** |
| 13.I am currently living alone. | 5 | 4 | 3 | 2 | 1 |
| 14.My family doesn't visit me often. | 5 | 4 | 3 | 2 | 1 |
| 15.I don't get along too well with my family. | 5 | 4 | 3 | 2 | 1 |
| 16.I feel that I'm becoming a burden to my family now. | 5 | 4 | 3 | 2 | 1 |
| 17.*My family provides help in a timely manner when I need it (reverse item). | 5 | 4 | 3 | 2 | 1 |
| 18.I feel that I am not recognized by my family. | 5 | 4 | 3 | 2 | 1 |
| 19.My family always fails to understand my thoughts. | 5 | 4 | 3 | 2 | 1 |
| 20.Caring for my family (spouse/children/ grandchildren, etc.) puts me under a lot of stress. | 5 | 4 | 3 | 2 | 1 |
|  | | | | | |
| **Dimension 3: Interpersonal level** | **Fully compliant** | **Compliant** | **Neutral** | **Not compliant** | **Not compliant at all** |
| 21.I have reduced the number of times I go out for activities. | 5 | 4 | 3 | 2 | 1 |
| 22.I now seldom contact others through phone, WeChat, and other means. | 5 | 4 | 3 | 2 | 1 |
| 23.I don't have friends I can ask for help. | 5 | 4 | 3 | 2 | 1 |
| 24.I am not satisfied with my current neighborhood relationships. | 5 | 4 | 3 | 2 | 1 |
| 25.I visit my family and friends less often. | 5 | 4 | 3 | 2 | 1 |
| 26.I rarely travel long distances by transport now (e.g. train/car). | 5 | 4 | 3 | 2 | 1 |
| 27.I don't usually participate in social activities on a regular basis (e.g., chess/ square dancing/religious activities, etc.). | 5 | 4 | 3 | 2 | 1 |
| 28.I don't have a social circle (e.g. recreational/fitness group) that I belong to right now. | 5 | 4 | 3 | 2 | 1 |
| 29.I attend fewer social events because of my mobility problems (e.g. travelling a long way/not physically able to do so). | 5 | 4 | 3 | 2 | 1 |
| 30.I'm worried about people gossiping about me. | 5 | 4 | 3 | 2 | 1 |
| 31.I'm less inclined to engage with strangers now. | 5 | 4 | 3 | 2 | 1 |
| 32.I would feel tired of dealing with people now. | 5 | 4 | 3 | 2 | 1 |
|  | | | | | |
| **Dimension 4: Community and social level** | **Fully compliant** | **Compliant** | **Neutral** | **Not compliant** | **Not compliant at all** |
| 33.I don't feel cared for by social organisations (e.g. neighbourhood councils/ volunteers etc.). | 5 | 4 | 3 | 2 | 1 |
| 34.I feel that community services do not adequately take into account the needs of the older adult (e.g. medical services/respect for the older adult activities). | 5 | 4 | 3 | 2 | 1 |
| 35.I feel that public facilities for the older adult are inadequate (e.g. fitness facilities/transport facilities). | 5 | 4 | 3 | 2 | 1 |
| 36.I feel that social policies provide good living security for the older adult (reverse item). | 5 | 4 | 3 | 2 | 1 |
| 37.I don't usually choose to travel by emerging modes of transport (e.g. metro/flight/internet car etc.). | 5 | 4 | 3 | 2 | 1 |
| 38.I often fail to understand how young people think. | 5 | 4 | 3 | 2 | 1 |
| 39.I feel like I don't fit in with the current social development. | 5 | 4 | 3 | 2 | 1 |
| 40.I don't learn new and emerging things to adapt to social development (e.g. learning to use a smartphone/ computer). | 5 | 4 | 3 | 2 | 1 |
| 41.I feel like I've lost my social status. | 5 | 4 | 3 | 2 | 1 |
| 42.I feel like I'm not contributing to society right now. | 5 | 4 | 3 | 2 | 1 |

**老年人社会衰弱评估量表初始版I**

**相比以往，最近一年您是否出现以下改变/处于以下情况？请您在符合的选项下打“√”。**

| **条目** | **完全**  **符合** | **符合** | **中立** | **不符合** | **完全**  **不符合** |
| --- | --- | --- | --- | --- | --- |
| **第一维度：个体层面** | | | | | |
| 1.我变得不太愿意讲话。 | 5 | 4 | 3 | 2 | 1 |
| 2.我现在常会感到孤独。 | 5 | 4 | 3 | 2 | 1 |
| 3.我没精力照顾好自己的日常生活起居。 | 5 | 4 | 3 | 2 | 1 |
| 4.我变得不那么在意自己的外在形象。 | 5 | 4 | 3 | 2 | 1 |
| 5.我常常感觉力不从心，有无用感。 | 5 | 4 | 3 | 2 | 1 |
| 6.我感觉每天的生活都很无趣。 | 5 | 4 | 3 | 2 | 1 |
| 7.我感觉没有人关心自己。 | 5 | 4 | 3 | 2 | 1 |
| 8.我有时会控制不住自己的情绪。 | 5 | 4 | 3 | 2 | 1 |
| 9.我现在对未来的生活没什么期待。 | 5 | 4 | 3 | 2 | 1 |
| 10.我每天不知道可以做些什么。 | 5 | 4 | 3 | 2 | 1 |
| 11.我对以前的兴趣爱好失去了热情。 | 5 | 4 | 3 | 2 | 1 |
| 12.我认为经济困难是影响我生活水平的重要因素。 | 5 | 4 | 3 | 2 | 1 |
|  | | | | | |
| **第二维度：家庭层面** | **完全**  **符合** | **符合** | **中立** | **不符合** | **完全**  **不符合** |
| 13.我目前处于独居状态。 | 5 | 4 | 3 | 2 | 1 |
| 14.我的家人不经常来探望我。 | 5 | 4 | 3 | 2 | 1 |
| 15.我和家人相处不太融洽。 | 5 | 4 | 3 | 2 | 1 |
| 16.我感觉现在自己成为了家里的拖累。 | 5 | 4 | 3 | 2 | 1 |
| 17.*在我需要时家人会及时提供帮助（反向条目）。 | 5 | 4 | 3 | 2 | 1 |
| 18.我感觉自己不被家人认可。 | 5 | 4 | 3 | 2 | 1 |
| 19.家人总是不能理解我的想法。 | 5 | 4 | 3 | 2 | 1 |
| 20.照顾家人（配偶/子孙等）让我承受了较大压力。 | 5 | 4 | 3 | 2 | 1 |
|  | | | | | |
| **第三维度：社交层面** | **完全**  **符合** | **符合** | **中立** | **不符合** | **完全**  **不符合** |
| 21.我外出活动的次数减少了。 | 5 | 4 | 3 | 2 | 1 |
| 22.我现在很少通过电话、微信等方式和别人联系。 | 5 | 4 | 3 | 2 | 1 |
| 23.我没有可以寻求帮助的朋友。 | 5 | 4 | 3 | 2 | 1 |
| 24.我对现在的邻里关系不满意。 | 5 | 4 | 3 | 2 | 1 |
| 25.我走亲访友的次数减少了。 | 5 | 4 | 3 | 2 | 1 |
| 26.我现在很少乘坐交通工具长途出行（如火车/汽车）。 | 5 | 4 | 3 | 2 | 1 |
| 27.我平时不经常参加社会活动（如下棋/广场舞/宗教活动等）。 | 5 | 4 | 3 | 2 | 1 |
| 28.我现在没有属于我的社交圈（如文娱/健身团体）。 | 5 | 4 | 3 | 2 | 1 |
| 29.我因为行动不便所以参加社交活动少（如路途远/身体状况不允许）。 | 5 | 4 | 3 | 2 | 1 |
| 30.我担心别人说关于我的闲言碎语。 | 5 | 4 | 3 | 2 | 1 |
| 31.我现在不太愿意和陌生人接触。 | 5 | 4 | 3 | 2 | 1 |
| 32.我现在会感觉和别人打交道很累。 | 5 | 4 | 3 | 2 | 1 |
|  | | | | | |
| **第四维度：社会层面** | **完全**  **符合** | **符合** | **中立** | **不符合** | **完全**  **不符合** |
| 33.我感觉不到社会组织对我的关心（如居委会/志愿者等）。 | 5 | 4 | 3 | 2 | 1 |
| 34.我感觉社区服务没充分考虑老年人需求（如医疗服务/敬老活动）。 | 5 | 4 | 3 | 2 | 1 |
| 35.我感觉针对老年人的社会公共设施不完善（如健身设施/交通设施）。 | 5 | 4 | 3 | 2 | 1 |
| 36.*我感觉社会政策给了老年人较好的生活保障（反向条目）。 | 5 | 4 | 3 | 2 | 1 |
| 37.我通常不会选择乘坐新兴的交通工具（如地铁/飞机/网约车等）。 | 5 | 4 | 3 | 2 | 1 |
| 38.我经常无法理解现在年轻人的想法。 | 5 | 4 | 3 | 2 | 1 |
| 39.我感觉自己不适应现在的社会发展。 | 5 | 4 | 3 | 2 | 1 |
| 40.我不会为了适应社会发展去学习新兴事物（如学习使用智能手机/电脑）。 | 5 | 4 | 3 | 2 | 1 |
| 41.我感觉自己的社会地位下降了。 | 5 | 4 | 3 | 2 | 1 |
| 42.我感觉现在的自己对社会没有贡献。 | 5 | 4 | 3 | 2 | 1 |

# Appendix 8: Item analysis

**Screening results of frequency analysis**

The difficulty and responsiveness of the items retrieved from the collected questionnaire were calculated. The response rate of each item fluctuanted from 87.55% to 100%, and the pass rate of each item was high. The concentration of each item option fluctuated between 29.43% and 67.55%, and did not exceed 80%, so all items were retained.

| Item | Item Pass Rate | Concentration of Choices | Item | Item Pass Rate | Concentration of Choices |
| --- | --- | --- | --- | --- | --- |
| 1 | 100.00% | 35.09% | 22 | 98.87% | 53.21% |
| 2 | 100.00% | 43.77% | 23 | 96.23% | 53.58% |
| 3 | 98.49% | 61.51% | 24 | 100.00% | 32.83% |
| 4 | 94.72% | 38.87% | 25 | 87.55% | 40.00% |
| 5 | 100.00% | 59.62% | 26 | 93.58% | 36.23% |
| 6 | 89.81% | 29.81% | 27 | 97.36% | 34.72% |
| 7 | 95.47% | 65.28% | 28 | 100.00% | 57.36% |
| 8 | 98.87% | 30.94% | 29 | 100.00% | 60.75% |
| 9 | 90.57% | 38.49% | 30 | 98.87% | 65.66% |
| 10 | 100.00% | 46.42% | 31 | 95.09% | 62.26% |
| 11 | 97.74% | 51.32% | 32 | 96.23% | 59.62% |
| 12 | 91.32% | 36.98% | 33 | 87.92% | 51.70% |
| 13 | 94.72% | 53.21% | 34 | 93.58% | 48.68% |
| 14 | 98.11% | 29.43% | 35 | 100.00% | 33.58% |
| 15 | 88.68% | 61.51% | 36 | 95.47% | 39.62% |
| 16 | 100.00% | 32.83% | 37 | 92.83% | 50.57% |
| 17 | 97.36% | 39.62% | 38 | 92.83% | 35.09% |
| 18 | 93.21% | 58.87% | 39 | 92.45% | 67.55% |
| 19 | 98.87% | 42.26% | 40 | 100.00% | 58.49% |
| 20 | 100.00% | 34.72% | 41 | 98.49% | 60.00% |
| 21 | 100.00% | 35.09% | 42 | 95.85% | 34.72% |

**Screening results of discrete trend method**

Upon calculation, it was found that the standard deviation (SD) of the items fluctuates between 0.771 and 1.455. Items with an SD less than 0.8 were considered not to meet the criteria. Items 7, 10, and 30 had an SD less than 0.8 and may be considered for removal.

| Item | Mean | SD | Item | Mean | SD |
| --- | --- | --- | --- | --- | --- |
| 1 | 2.83 | 1.308 | 22 | 2.74 | 1.339 |
| 2 | 2.78 | 1.259 | 23 | 2.65 | 1.252 |
| 3 | 2.67 | 1.155 | 24 | 2.74 | 1.255 |
| 4 | 2.74 | 1.233 | 25 | 2.71 | 1.303 |
| 5 | 2.59 | 1.197 | 26 | 2.91 | 1.264 |
| 6 | 2.81 | 1.281 | 27 | 2.68 | 1.224 |
| 7 | 2.48 | 0.788 | 28 | 2.66 | 1.251 |
| 8 | 2.71 | 1.241 | 29 | 2.66 | 1.291 |
| 9 | 2.78 | 1.289 | 30 | 2.61 | 0.771 |
| 10 | 2.56 | 0.781 | 31 | 2.63 | 1.246 |
| 11 | 2.87 | 1.318 | 32 | 2.75 | 1.282 |
| 12 | 2.94 | 1.380 | 33 | 2.83 | 1.455 |
| 13 | 2.65 | 1.264 | 34 | 2.76 | 1.399 |
| 14 | 2.58 | 1.223 | 35 | 2.75 | 1.314 |
| 15 | 2.72 | 1.255 | 36 | 2.75 | 1.298 |
| 16 | 2.57 | 1.217 | 37 | 2.82 | 1.247 |
| 17 | 2.45 | 1.121 | 38 | 2.67 | 0.919 |
| 18 | 2.62 | 1.178 | 39 | 2.69 | 1.341 |
| 19 | 2.71 | 1.229 | 40 | 2.60 | 1.282 |
| 20 | 2.69 | 1.274 | 41 | 2.63 | 1.379 |
| 21 | 2.96 | 1.318 | 42 | 2.75 | 1.438 |

**Screening results of critical ratio method**

After arranging the total scores of 265 questionnaires from low to high, individuals in the top 27% of the total score were categorized as the high score group, and those in the bottom 27% as the low score group. The mean scores for each item were calculated for both groups. A t-test was employed to compare the differences between the two groups on each item. With a P-value of less than 0.05 for all 42 items, significant differences were indicated, suggesting that the items have good discriminative power. Consequently, no items were removed.

| Item | High Score Group | Low Score Group | *t* | *P* |
| --- | --- | --- | --- | --- |
| 1 | 3.68±1.149 | 1.81±0.882 | 10.985 | 0.000 |
| 2 | 3.51±1.061 | 1.83±0.904 | 10.229 | 0.000 |
| 3 | 3.24±1.132 | 1.97±0.903 | 7.405 | 0.000 |
| 4 | 3.38±1.093 | 1.88±0.978 | 8.678 | 0.000 |
| 5 | 3.18±1.237 | 1.86±0.877 | 7.383 | 0.000 |
| 6 | 3.50±1.126 | 1.86±0.893 | 9.677 | 0.000 |
| 7 | 2.72±0.843 | 2.24±0.593 | 4.002 | 0.000 |
| 8 | 3.40±1.171 | 1.83±0.839 | 9.245 | 0.000 |
| 9 | 3.65±1.165 | 1.81±0.898 | 10.657 | 0.000 |
| 10 | 2.64±0.737 | 2.26±0.581 | 3.389 | 0.001 |
| 11 | 3.63±1.106 | 1.83±0.872 | 10.793 | 0.000 |
| 12 | 3.72±1.165 | 1.82±0.877 | 11.068 | 0.000 |
| 13 | 3.38±1.227 | 2.10±1.103 | 6.573 | 0.000 |
| 14 | 3.49±1.138 | 1.94±0.963 | 8.776 | 0.000 |
| 15 | 3.26±1.199 | 2.31±1.146 | 4.903 | 0.000 |
| 16 | 3.63±1.119 | 1.86±0.861 | 10.603 | 0.000 |
| 17 | 3.18±1.142 | 1.86±0.756 | 8.172 | 0.000 |
| 18 | 3.49±1.061 | 1.86±0.861 | 10.09 | 0.000 |
| 19 | 3.60±0.944 | 2.10±0.995 | 9.277 | 0.000 |
| 20 | 3.67±1.061 | 1.93±0.939 | 10.393 | 0.000 |
| 21 | 3.63±1.261 | 2.53±1.175 | 5.403 | 0.000 |
| 22 | 3.96±0.971 | 1.65±0.754 | 15.922 | 0.000 |
| 23 | 3.64±1.117 | 1.74±0.856 | 11.473 | 0.000 |
| 24 | 3.61±1.082 | 1.92±1.097 | 9.33 | 0.000 |
| 25 | 3.96±0.971 | 1.72±0.826 | 14.888 | 0.000 |
| 26 | 3.22±1.270 | 2.75±1.207 | 2.287 | 0.024 |
| 27 | 3.42±1.172 | 2.14±1.092 | 6.77 | 0.000 |
| 28 | 3.71±1.067 | 1.75±0.915 | 11.818 | 0.000 |
| 29 | 3.86±1.025 | 1.53±0.691 | 16.012 | 0.000 |
| 30 | 2.75±0.783 | 2.49±0.805 | 1.995 | 0.048 |
| 31 | 3.63±1.106 | 1.61±0.703 | 13.037 | 0.000 |
| 32 | 3.58±1.110 | 1.78±0.876 | 10.835 | 0.000 |
| 33 | 4.14±0.997 | 1.72±0.923 | 15.093 | 0.000 |
| 34 | 3.79±1.100 | 1.94±1.124 | 9.965 | 0.000 |
| 35 | 3.76±0.986 | 1.96±0.911 | 11.417 | 0.000 |
| 36 | 3.74±1.035 | 1.82±0.893 | 11.899 | 0.000 |
| 37 | 3.21±1.255 | 2.57±1.231 | 3.083 | 0.002 |
| 38 | 2.93±0.861 | 2.38±0.895 | 3.795 | 0.000 |
| 39 | 3.74±1.113 | 1.79±0.978 | 11.136 | 0.000 |
| 40 | 3.46±1.087 | 1.94±1.149 | 8.121 | 0.000 |
| 41 | 3.68±1.254 | 1.86±1.066 | 9.382 | 0.000 |
| 42 | 3.94±1.086 | 1.68±1.019 | 12.9 | 0.000 |

**Screening results of correlation coefficient method**

The analysis of the correlation between each item and the total score revealed that items 7, 10, 15, 21, 26, 30, 37, and 38 had correlation coefficients less than 0.4 with the total score, and thus, they are considered for removal.

| Item | Correlation Coefficient with Total Score | *P* | Item | Correlation Coefficient with Total Score | *P* |
| --- | --- | --- | --- | --- | --- |
| 1 | 0.573 | ＜0.001 | 22 | 0.674 | ＜0.001 |
| 2 | 0.529 | ＜0.001 | 23 | 0.612 | ＜0.001 |
| 3 | 0.428 | ＜0.001 | 24 | 0.536 | ＜0.001 |
| 4 | 0.472 | ＜0.001 | 25 | 0.679 | ＜0.001 |
| 5 | 0.451 | ＜0.001 | 26 | 0.363 | 0.008 |
| 6 | 0.512 | ＜0.001 | 27 | 0.429 | ＜0.001 |
| 7 | 0.263 | ＜0.001 | 28 | 0.613 | ＜0.001 |
| 8 | 0.481 | ＜0.001 | 29 | 0.694 | ＜0.001 |
| 9 | 0.579 | ＜0.001 | 30 | 0.124 | 0.043 |
| 10 | 0.202 | 0.001 | 31 | 0.627 | ＜0.001 |
| 11 | 0.541 | ＜0.001 | 32 | 0.538 | ＜0.001 |
| 12 | 0.552 | ＜0.001 | 33 | 0.679 | ＜0.001 |
| 13 | 0.405 | ＜0.001 | 34 | 0.551 | ＜0.001 |
| 14 | 0.505 | ＜0.001 | 35 | 0.542 | ＜0.001 |
| 15 | 0.322 | ＜0.001 | 36 | 0.608 | ＜0.001 |
| 16 | 0.553 | ＜0.001 | 37 | 0.216 | ＜0.001 |
| 17 | 0.471 | ＜0.001 | 38 | 0.226 | ＜0.001 |
| 18 | 0.529 | ＜0.001 | 39 | 0.580 | ＜0.001 |
| 19 | 0.512 | ＜0.001 | 40 | 0.461 | ＜0.001 |
| 20 | 0.526 | ＜0.001 | 41 | 0.548 | ＜0.001 |
| 21 | 0.327 | ＜0.001 | 42 | 0.624 | ＜0.001 |

**Screening results of Cronbach's coefficient method**

The statistical analysis yielded a Cronbach's α of 0.925 for the scale, indicating good internal consistency. Upon the exclusion of items 10, 26, 30, 37, and 38, the Cronbach's α for the scale increased, suggesting that these items were considered for deletion.

| Item | α | Item | α |
| --- | --- | --- | --- |
| 1 | 0.923 | 22 | 0.922 |
| 2 | 0.923 | 23 | 0.922 |
| 3 | 0.924 | 24 | 0.923 |
| 4 | 0.924 | 25 | 0.921 |
| 5 | 0.924 | 26 | 0.927 |
| 6 | 0.923 | 27 | 0.924 |
| 7 | 0.925 | 28 | 0.922 |
| 8 | 0.924 | 29 | 0.921 |
| 9 | 0.923 | 30 | 0.926 |
| 10 | 0.926 | 31 | 0.922 |
| 11 | 0.923 | 32 | 0.923 |
| 12 | 0.923 | 33 | 0.921 |
| 13 | 0.924 | 34 | 0.923 |
| 14 | 0.923 | 35 | 0.923 |
| 15 | 0.925 | 36 | 0.922 |
| 16 | 0.923 | 37 | 0.926 |
| 17 | 0.924 | 38 | 0.926 |
| 18 | 0.923 | 39 | 0.923 |
| 19 | 0.923 | 40 | 0.924 |
| 20 | 0.923 | 41 | 0.923 |
| 21 | 0.925 | 42 | 0.922 |

Considering the various statistical results from the item analysis, the principle for item selection was to retain items indicated by at least three methods in the item analysis, and to eliminate items that failed to meet the criteria in two or more methods. Consequently, items 7, 10, 26, 30, 37, and 38 were removed. 3. Consequently, a scale consisting of 36 items across 4 dimensions was constructed.

| Item | Frequency Analysis | Discrete Trend Method | Critical Ratio Method | Correlation Coefficient Method | Cronbach's coefficient method | Selection Results |
| --- | --- | --- | --- | --- | --- | --- |
| 1 | √ | √ | √ | √ | √ | √ |
| 2 | √ | √ | √ | √ | √ | √ |
| 3 | √ | √ | √ | √ | √ | √ |
| 4 | √ | √ | √ | √ | √ | √ |
| 5 | √ | √ | √ | √ | √ | √ |
| 6 | √ | √ | √ | √ | √ | √ |
| 7 | √ | × | √ | × | √ | × |
| 8 | √ | √ | √ | √ | √ | √ |
| 9 | √ | √ | √ | √ | √ | √ |
| 10 | √ | × | √ | × | × | × |
| 11 | √ | √ | √ | √ | √ | √ |
| 12 | √ | √ | √ | √ | √ | √ |
| 13 | √ | √ | √ | √ | √ | √ |
| 14 | √ | √ | √ | √ | √ | √ |
| 15 | √ | √ | √ | × | √ | √ |
| 16 | √ | √ | √ | √ | √ | √ |
| 17 | √ | √ | √ | √ | √ | √ |
| 18 | √ | √ | √ | √ | √ | √ |
| 19 | √ | √ | √ | √ | √ | √ |
| 20 | √ | √ | √ | √ | √ | √ |
| 21 | √ | √ | √ | × | √ | √ |
| 22 | √ | √ | √ | √ | √ | √ |
| 23 | √ | √ | √ | √ | √ | √ |
| 24 | √ | √ | √ | √ | √ | √ |
| 25 | √ | √ | √ | √ | √ | √ |
| 26 | √ | √ | √ | × | × | × |
| 27 | √ | √ | √ | √ | √ | √ |
| 28 | √ | √ | √ | √ | √ | √ |
| 29 | √ | √ | √ | √ | √ | √ |
| 30 | √ | × | √ | × | × | × |
| 31 | √ | √ | √ | √ | √ | √ |
| 32 | √ | √ | √ | √ | √ | √ |
| 33 | √ | √ | √ | √ | √ | √ |
| 34 | √ | √ | √ | √ | √ | √ |
| 35 | √ | √ | √ | √ | √ | √ |
| 36 | √ | √ | √ | √ | √ | √ |
| 37 | √ | √ | √ | × | × | × |
| 38 | √ | √ | √ | × | × | × |
| 39 | √ | √ | √ | √ | √ | √ |
| 40 | √ | √ | √ | √ | √ | √ |
| 41 | √ | √ | √ | √ | √ | √ |
| 42 | √ | √ | √ | √ | √ | √ |

# Appendix 9: CVSFS 2.0 version

Compared to the past, have you experienced any of the following changes/been in any of the following situations in the last year? Please put a check mark under the options that match.

| **Scale entry** | **Fully compliant** | **Compliant** | **Neutral** | **Not compliant** | **Not compliant at all** |
| --- | --- | --- | --- | --- | --- |
| **Dimension 1: Individual level** | | | | | |
| 1.I've become very reluctant to talk. | 5 | 4 | 3 | 2 | 1 |
| 2.I often feel lonely now. | 5 | 4 | 3 | 2 | 1 |
| 3.I don't have the energy to take care of my daily routine. | 5 | 4 | 3 | 2 | 1 |
| 4.I became less concerned with my outward appearance. | 5 | 4 | 3 | 2 | 1 |
| 5.I often feel overwhelmed and have a sense of uselessness. | 5 | 4 | 3 | 2 | 1 |
| 6.I feel like every day is boring. | 5 | 4 | 3 | 2 | 1 |
| 7.I can't control my emotions sometimes. | 5 | 4 | 3 | 2 | 1 |
| 8.I don't have much to look forward to in the future right now. | 5 | 4 | 3 | 2 | 1 |
| 9.I lost my enthusiasm for my former hobbies. | 5 | 4 | 3 | 2 | 1 |
| 10.I think financial hardship is a major factor in my standard of living. | 5 | 4 | 3 | 2 | 1 |
|  | | | | | |
| **Dimension 2: family level** | **Fully compliant** | **Compliant** | **Neutral** | **Not compliant** | **Not compliant at all** |
| 11.I am currently living alone. | 5 | 4 | 3 | 2 | 1 |
| 12.My family doesn't visit me often. | 5 | 4 | 3 | 2 | 1 |
| 13.I don't get along too well with my family. | 5 | 4 | 3 | 2 | 1 |
| 14.I feel that I'm becoming a burden to my family now. | 5 | 4 | 3 | 2 | 1 |
| 15.*My family provides help in a timely manner when I need it (reverse item). | 5 | 4 | 3 | 2 | 1 |
| 16.I feel that I am not recognized by my family. | 5 | 4 | 3 | 2 | 1 |
| 17.My family always fails to understand my thoughts. | 5 | 4 | 3 | 2 | 1 |
| 18.Caring for my family (spouse/children/ grandchildren, etc.) puts me under a lot of stress. | 5 | 4 | 3 | 2 | 1 |
|  | | | | | |
| **Dimension 3: Interpersonal level** | **Fully compliant** | **Compliant** | **Neutral** | **Not compliant** | **Not compliant at all** |
| 19.I have reduced the number of times I go out for activities. | 5 | 4 | 3 | 2 | 1 |
| 20.I now seldom contact others through phone, WeChat, and other means. | 5 | 4 | 3 | 2 | 1 |
| 21.I don't have friends I can ask for help. | 5 | 4 | 3 | 2 | 1 |
| 22.I am not satisfied with my current neighborhood relationships. | 5 | 4 | 3 | 2 | 1 |
| 23.I visit my family and friends less often. | 5 | 4 | 3 | 2 | 1 |
| 24.I don't usually participate in social activities on a regular basis (e.g., chess/ square dancing/religious activities, etc.). | 5 | 4 | 3 | 2 | 1 |
| 25.I don't have a social circle (e.g. recreational/fitness group) that I belong to right now. | 5 | 4 | 3 | 2 | 1 |
| 26.I attend fewer social events because of my mobility problems (e.g. travelling a long way/not physically able to do so). | 5 | 4 | 3 | 2 | 1 |
| 27.I'm less inclined to engage with strangers now. | 5 | 4 | 3 | 2 | 1 |
| 28.I would feel tired when dealing with other people now. | 5 | 4 | 3 | 2 | 1 |
|  | | | | | |
| **Dimension 4: Community and social level** | **Fully compliant** | **Compliant** | **Neutral** | **Not compliant** | **Not compliant at all** |
| 29.I don't feel cared for by social organisations (e.g. neighbourhood councils/ volunteers etc.). | 5 | 4 | 3 | 2 | 1 |
| 30.I feel that community services do not adequately take into account the needs of the older adult (e.g. medical services/respect for the older adult activities). | 5 | 4 | 3 | 2 | 1 |
| 31.I feel that public facilities for the older adult are inadequate (e.g. fitness facilities/transport facilities). | 5 | 4 | 3 | 2 | 1 |
| 1. *I feel that social policies provide good living security for the older adult (reverse item). | 5 | 4 | 3 | 2 | 1 |
| 33.I feel like I don't fit in with the current social development. | 5 | 4 | 3 | 2 | 1 |
| 34.I don't learn new and emerging things to adapt to social development (e.g. learning to use a smartphone/ computer). | 5 | 4 | 3 | 2 | 1 |
| 35.I feel like I've lost my social status. | 5 | 4 | 3 | 2 | 1 |
| 36.I feel like I'm not contributing to society right now. | 5 | 4 | 3 | 2 | 1 |

**老年人社会衰弱评估量表初始版**Ⅱ**（测试版）**

**相比以往，最近一年您是否出现以下改变/处于以下情况？请您在符合的选项下打“√”。**

| **条目** | **完全**  **符合** | **符合** | **中立** | **不符合** | **完全**  **不符合** |
| --- | --- | --- | --- | --- | --- |
| **第一维度：个体层面** | | | | | |
| 1.我变得不太愿意讲话。 | 5 | 4 | 3 | 2 | 1 |
| 2.我现在常会感到孤独。 | 5 | 4 | 3 | 2 | 1 |
| 3.我没精力照顾好自己的日常生活起居。 | 5 | 4 | 3 | 2 | 1 |
| 4.我变得不那么在意自己的外在形象。 | 5 | 4 | 3 | 2 | 1 |
| 5.我常常感觉力不从心，有无用感。 | 5 | 4 | 3 | 2 | 1 |
| 6.我感觉每天的生活都很无趣。 | 5 | 4 | 3 | 2 | 1 |
| 7.我有时会控制不住自己的情绪。 | 5 | 4 | 3 | 2 | 1 |
| 8.我现在对未来的生活没什么期待。 | 5 | 4 | 3 | 2 | 1 |
| 9.我对以前的兴趣爱好失去了热情。 | 5 | 4 | 3 | 2 | 1 |
| 10.我认为经济困难是影响我生活水平的重要因素。 | 5 | 4 | 3 | 2 | 1 |
|  | | | | | |
| **第二维度：家庭层面** | **完全**  **符合** | **符合** | **中立** | **不符合** | **完全**  **不符合** |
| 11.我目前处于独居状态。 | 5 | 4 | 3 | 2 | 1 |
| 12.我的家人不经常来探望我。 | 5 | 4 | 3 | 2 | 1 |
| 13.我和家人相处不太融洽。 | 5 | 4 | 3 | 2 | 1 |
| 14.我感觉现在自己成为了家里的拖累。 | 5 | 4 | 3 | 2 | 1 |
| 15.*在我需要时家人会及时提供帮助（反向条目）。 | 5 | 4 | 3 | 2 | 1 |
| 16.我感觉自己不被家人认可。 | 5 | 4 | 3 | 2 | 1 |
| 17.家人总是不能理解我的想法。 | 5 | 4 | 3 | 2 | 1 |
| 18.照顾家人（配偶/子孙等）让我承受了较大压力。 | 5 | 4 | 3 | 2 | 1 |
|  | | | | | |
| **第三维度：社交层面** | **完全**  **符合** | **符合** | **中立** | **不符合** | **完全**  **不符合** |
| 19.我外出活动的次数减少了。 | 5 | 4 | 3 | 2 | 1 |
| 20.我现在很少通过电话、微信等方式和别人联系。 | 5 | 4 | 3 | 2 | 1 |
| 21.我没有可以寻求帮助的朋友。 | 5 | 4 | 3 | 2 | 1 |
| 22.我对现在的邻里关系不满意。 | 5 | 4 | 3 | 2 | 1 |
| 23.我走亲访友的次数减少了。 | 5 | 4 | 3 | 2 | 1 |
| 24.我平时不经常参加社交活动（如下棋/广场舞/宗教活动等）。 | 5 | 4 | 3 | 2 | 1 |
| 25.我没有属于我的社交圈（如文娱/健身团体）。 | 5 | 4 | 3 | 2 | 1 |
| 26.我因为行动不便所以参加社交活动少（如路途远/身体状况不允许）。 | 5 | 4 | 3 | 2 | 1 |
| 27.我现在不太愿意和陌生人接触。 | 5 | 4 | 3 | 2 | 1 |
| 28.我现在会感觉和别人打交道很累。 | 5 | 4 | 3 | 2 | 1 |
|  | | | | | |
| **第四维度：社会层面** | **完全**  **符合** | **符合** | **中立** | **不符合** | **完全**  **不符合** |
| 29.我感觉不到社会组织对我的关心（如居委会/志愿者等）。 | 5 | 4 | 3 | 2 | 1 |
| 30.我感觉社区服务没充分考虑老年人需求（如医疗服务/敬老活动）。 | 5 | 4 | 3 | 2 | 1 |
| 31.我感觉针对老年人的社会公共设施不完善（如健身设施/交通设施）。 | 5 | 4 | 3 | 2 | 1 |
| 32.*我感觉社会政策给了老年人较好的生活保障（反向条目）。 | 5 | 4 | 3 | 2 | 1 |
| 33.我感觉自己不适应现在的社会发展。 | 5 | 4 | 3 | 2 | 1 |
| 34.我没有为了适应社会发展去学习新兴事物（如学习使用智能手机/电脑）。 | 5 | 4 | 3 | 2 | 1 |
| 35.我感觉自己的社会地位下降了。 | 5 | 4 | 3 | 2 | 1 |
| 36.我感觉现在的自己对社会没有贡献。 | 5 | 4 | 3 | 2 | 1 |

# Appendix 10

| ROC curvilinear coordinates | | | |
| --- | --- | --- | --- |
| Test result variable: SFAS 2.0 score | sensitivity | 1 - specificity | YI |
| 44 | 1 | 1 | 0 |
| 45.5 | 1 | 0.995 | 0.005 |
| 48 | 1 | 0.991 | 0.009 |
| 51 | 1 | 0.986 | 0.014 |
| 53.5 | 1 | 0.982 | 0.018 |
| 55.5 | 1 | 0.973 | 0.027 |
| 56.5 | 1 | 0.959 | 0.041 |
| 57.5 | 1 | 0.95 | 0.05 |
| 58.5 | 1 | 0.932 | 0.068 |
| 60.5 | 1 | 0.923 | 0.077 |
| 62.5 | 1 | 0.895 | 0.105 |
| 63.5 | 1 | 0.882 | 0.118 |
| 64.5 | 1 | 0.873 | 0.127 |
| 65.5 | 1 | 0.864 | 0.136 |
| 66.5 | 1 | 0.855 | 0.145 |
| 67.5 | 1 | 0.85 | 0.15 |
| 68.5 | 1 | 0.836 | 0.164 |
| 69.5 | 0.978 | 0.827 | 0.151 |
| 70.5 | 0.978 | 0.8 | 0.178 |
| 71.5 | 0.978 | 0.795 | 0.183 |
| 72.5 | 0.978 | 0.782 | 0.196 |
| 73.5 | 0.978 | 0.773 | 0.205 |
| 74.5 | 0.978 | 0.764 | 0.214 |
| 75.5 | 0.978 | 0.755 | 0.223 |
| 76.5 | 0.978 | 0.736 | 0.242 |
| 77.5 | 0.978 | 0.727 | 0.251 |
| 78.5 | 0.978 | 0.718 | 0.26 |
| 79.5 | 0.978 | 0.709 | 0.269 |
| 80.5 | 0.978 | 0.695 | 0.283 |
| 81.5 | 0.978 | 0.682 | 0.296 |
| 82.5 | 0.978 | 0.668 | 0.31 |
| 83.5 | 0.978 | 0.664 | 0.314 |
| 84.5 | 0.956 | 0.632 | 0.324 |
| 85.5 | 0.956 | 0.605 | 0.351 |
| 86.5 | 0.956 | 0.586 | 0.37 |
| 87.5 | 0.956 | 0.577 | 0.379 |
| 88.5 | 0.956 | 0.564 | 0.392 |
| 89.5 | 0.956 | 0.541 | 0.415 |
| 90.5 | 0.933 | 0.509 | 0.424 |
| 91.5 | 0.933 | 0.486 | 0.447 |
| 92.5 | 0.933 | 0.468 | 0.465 |
| 93.5 | 0.933 | 0.445 | 0.488 |
| 94.5 | 0.933 | 0.418 | 0.515 |
| 95.5 | 0.933 | 0.409 | 0.524 |
| 96.5 | 0.911 | 0.395 | 0.516 |
| 97.5 | 0.911 | 0.382 | 0.529 |
| 98.5 | 0.889 | 0.373 | 0.516 |
| 99.5 | 0.889 | 0.364 | 0.525 |
| 100.5 | 0.867 | 0.332 | 0.535 |
| 101.5 | 0.867 | 0.327 | 0.54 |
| 102.5 | 0.844 | 0.314 | 0.53 |
| 103.5 | 0.844 | 0.291 | 0.553 |
| 104.5 | 0.844 | 0.277 | 0.567 |
| 105.5 | 0.822 | 0.255 | 0.567 |
| 106.5 | 0.822 | 0.241 | 0.581 |
| 107.5 | 0.822 | 0.232 | 0.59 |
| 108.5 | 0.822 | 0.223 | 0.599 |
| 109.5 | 0.8 | 0.218 | 0.582 |
| 110.5 | 0.8 | 0.2 | 0.6 |
| 111.5 | 0.778 | 0.195 | 0.583 |
| 112.5 | 0.778 | 0.191 | 0.587 |
| 113.5 | 0.778 | 0.186 | 0.592 |
| 114.5 | 0.778 | 0.177 | 0.601 |
| 115.5 | 0.778 | 0.168 | 0.61 |
| 116.5 | 0.756 | 0.155 | 0.601 |
| 117.5 | 0.756 | 0.15 | 0.606 |
| 118.5 | 0.733 | 0.145 | 0.588 |
| 119.5 | 0.711 | 0.132 | 0.579 |
| 120.5 | 0.689 | 0.127 | 0.562 |
| 121.5 | 0.689 | 0.118 | 0.571 |
| 122.5 | 0.622 | 0.091 | 0.531 |
| 123.5 | 0.6 | 0.082 | 0.518 |
| 125 | 0.578 | 0.077 | 0.501 |
| 126.5 | 0.556 | 0.073 | 0.483 |
| 128 | 0.511 | 0.073 | 0.438 |
| 129.5 | 0.489 | 0.059 | 0.43 |
| 130.5 | 0.444 | 0.055 | 0.389 |
| 132 | 0.422 | 0.05 | 0.372 |
| 133.5 | 0.378 | 0.041 | 0.337 |
| 134.5 | 0.333 | 0.032 | 0.301 |
| 135.5 | 0.289 | 0.027 | 0.262 |
| 136.5 | 0.244 | 0.018 | 0.226 |
| 137.5 | 0.2 | 0.014 | 0.186 |
| 138.5 | 0.2 | 0.009 | 0.191 |
| 140 | 0.178 | 0.009 | 0.169 |
| 141.5 | 0.156 | 0.005 | 0.151 |
| 143 | 0.156 | 0 | 0.156 |
| 146 | 0.133 | 0 | 0.133 |
| 148.5 | 0.111 | 0 | 0.111 |
| 152 | 0.089 | 0 | 0.089 |
| 155.5 | 0.067 | 0 | 0.067 |
| 156.5 | 0.044 | 0 | 0.044 |
| 159.5 | 0.022 | 0 | 0.022 |
| 163 | 0 | 0 | 0 |

# Appendix 11

| Expert rating scale of content validity index | | | | | |
| --- | --- | --- | --- | --- | --- |
| item | number of experts with a score of 3 or 4 | I-CVI | item | number of experts with a score of 3 or 4 | I-CVI |
| individual level |  |  | interpersonal level |  |  |
| 1 | 9 | 1.000 | 19 | 9 | 1.000 |
| 2 | 9 | 1.000 | 20 | 9 | 1.000 |
| 3 | 9 | 1.000 | 21 | 8 | 0.889 |
| 4 | 8 | 0.889 | 22 | 9 | 1.000 |
| 5 | 9 | 1.000 | 23 | 9 | 1.000 |
| 6 | 8 | 0.889 | 24 | 9 | 1.000 |
| 7 | 9 | 1.000 | 25 | 9 | 1.000 |
| 8 | 9 | 1.000 | 26 | 9 | 1.000 |
| 9 | 9 | 1.000 | 27 | 9 | 1.000 |
| 10 | 9 | 1.000 | 28 | 9 | 1.000 |
| family level |  |  | community and social level |  |  |
| 11 | 9 | 1.000 | 29 | 9 | 1.000 |
| 12 | 8 | 0.889 | 30 | 9 | 1.000 |
| 13 | 9 | 1.000 | 31 | 9 | 1.000 |
| 14 | 9 | 1.000 | 32 | 9 | 1.000 |
| 15 | 9 | 1.000 | 33 | 9 | 1.000 |
| 16 | 9 | 1.000 | 34 | 9 | 1.000 |
| 17 | 8 | 0.889 | 35 | 8 | 0.889 |
| 18 | 9 | 1.000 | 36 | 9 | 1.000 |

# Appendix 12

| Opinions of participant on content validity index | | | | |
| --- | --- | --- | --- | --- |
| Question content | Opinions of participant | | | Proportion of positive opinions |
|  | yes | neutral | no |  |
| Question 1：Is the questionnaire comprehensive in its coverage of social frailty? | 50 | 0 | 0 | 100% |
| Question 2：Is the questionnaire comprehensive in its coverage of social frailty?’ | 47 | 3 | 0 | 94% |
| Question 3：Does the scale help you to judge whether or not a person is social frailty? | 48 | 2 | 0 | 96% |

# Appendix 13


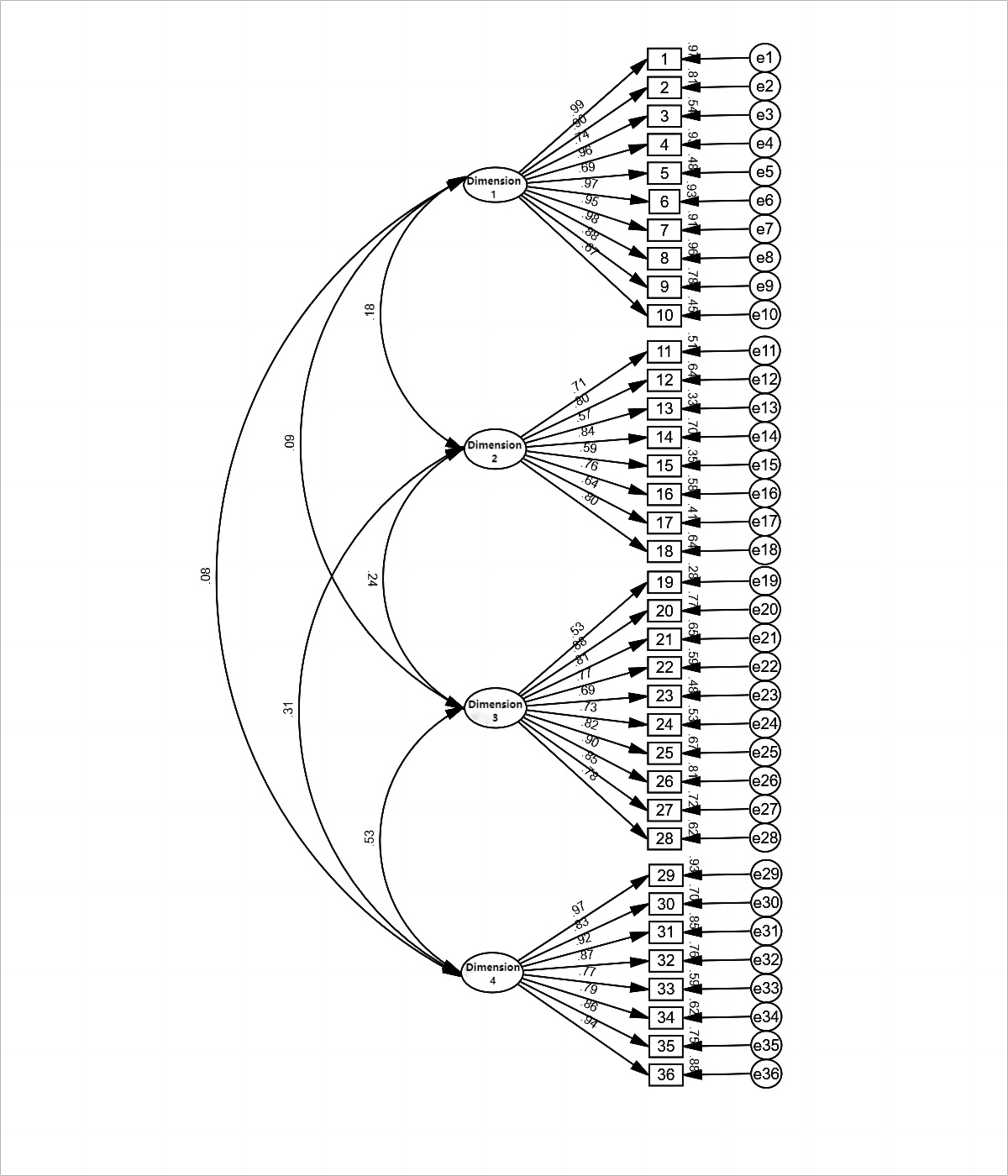


Structural equation model diagram of SFAS 2.0 version. Dimension 1, individual level; Dimension 2, family level; Dimension 3, interpersonal level; Dimension 4, community and social level. Numbers 1~36 are scale items 1 ~ 36; e1~e36 are residuals.
